# MUC1 deficiency mediates corticosteroid resistance in chronic obstructive pulmonary disease

**DOI:** 10.1186/s12931-018-0927-4

**Published:** 2018-11-20

**Authors:** Javier Milara, Lucía Díaz-Platas, Sonia Contreras, Pilar Ribera, Inés Roger, Beatriz Ballester, Paula Montero, Ángel Cogolludo, Esteban Morcillo, Julio Cortijo

**Affiliations:** 1 Department of Pharmacology, Faculty of Medicine, Jaume I University, Castellón de la Plana, Spain; 2 Pharmacy Unit, University General Hospital Consortium, Valencia, Spain; 3 CIBERES, Health Institute Carlos III, Valencia, Spain; 4 Unidade Radiofármacos PET, GALARIA, Santiago de Compostela, A Coruña, Spain; 5 Department of Pharmacology, Faculty of Medicine, University of Valencia, Valencia, Spain; 6 Department of Pharmacology, School of Medicine, Universidad Complutense de Madrid, Madrid, Spain; 7 Health Research Institute INCLIVA, Valencia, Spain; 8 Research and teaching Unit, University General Hospital Consortium, Valencia, Spain; 9 Unidad de Investigación Clínica, Consorcio Hospital General Universitario, Avenida tres cruces s/n, E-46014 Valencia, Spain

**Keywords:** Chronic obstructive pulmonary disease, MUC1, Corticosteroid resistance

## Abstract

**Background:**

Lung inflammation in COPD is poorly controlled by inhaled corticosteroids (ICS). Strategies to improve ICS efficacy or the search of biomarkers who may select those patients candidates to receive ICS in COPD are needed. Recent data indicate that MUC1 cytoplasmic tail (CT) membrane mucin can mediate corticosteroid efficacy in chronic rhinosinusitis. The objective of this work was to analyze the previously unexplored role of MUC1 on corticosteroid efficacy in COPD in vitro and in vivo models.

**Methods:**

MUC1-CT expression was measured by real time PCR, western blot, immunohistochemistry and immunofluorescence. The inflammatory mediators IL-8, MMP9, GM-CSF and MIP3α were measured by ELISA. The effect of MUC1 on inflammation and corticosteroid anti-inflammatory effects was measured using cell siRNA in vitro and Muc1-KO in vivo animal models.

**Results:**

MUC1-CT expression was downregulated in lung tissue, bronchial epithelial cells and lung neutrophils from smokers (*n* = 11) and COPD (n = 11) patients compared with healthy subjects (*n* = 10). MUC1 was correlated with FEV1% (ρ = 0.7479; *p* < 0.0001) in smokers and COPD patients. Cigarette smoke extract (CSE) decreased the expression of MUC1 and induced corticosteroid resistance in human primary bronchial epithelial cells and human neutrophils. MUC1 Gene silencing using siRNA-MUC1 impaired the anti-inflammatory effects of dexamethasone and reduced glucocorticoid response element activation. Dexamethasone promoted glucocorticoid receptor alpha (GRα) and MUC1-CT nuclear translocation and co-localization that was inhibited by CSE. Lung function decline and inflammation induced by lipopolysaccharide and cigarette smoke in Muc1 KO mice was resistant to dexamethasone.

**Conclusions:**

These results confirm a role for MUC1-CT mediating corticosteroid efficacy in COPD.

**Electronic supplementary material:**

The online version of this article (10.1186/s12931-018-0927-4) contains supplementary material, which is available to authorized users.

## Background

Chronic obstructive pulmonary disease (COPD) is a chronic inflammatory, progressive and debilitating lung disease. It is a major cause of chronic morbidity, the fourth leading cause of death worldwide and is projected to be the third leading cause of death by 2020 [1]. The primary cause of COPD is chronic exposure to cigarette smoke, which leads to airway inflammation and remodeling, thus increasing airflow limitation. In contrast with the magnitude of COPD, therapeutic options remains limited, and none of the available drugs have demonstrated to reduce lung function decline and mortality. The current first-line maintenance treatment for COPD involves the use of bronchodilators, including long-acting muscarinic antagonists (LAMAs) and long-acting beta 2 agonists (LABAs). Combination treatment with LAMA and LABA improves respiratory function, reduces symptoms and exacerbations compared to monotherapy or inhaled corticosteroids (ICS)/LABA combinations. Anti-inflammatory options for COPD are limited to corticosteroids and phosphodiesterase 4 inhibitors. While inhaled corticosteroids (ICS) have shown good activity in asthma, they are much less effective in improving lung function and have little or no effect on controlling the underlying chronic inflammation in COPD patients [2]. Triple therapy based on ICS in combination with LABAs and LAMAs is indicated in severe COPD at risk of exacerbations [3], however the use of ICS is under debate and needs more evidence to adequately select the COPD phenotype that takes advantage of this combination.

The loss of corticosteroid anti-inflammatory efficacy is a characteristic feature in COPD patients. Several molecular mechanisms of corticosteroid resistance have now been identified involving phosphorylation and modifications of glucocorticoid receptor (GR), and loss of histone deacetylase-2 (HDAC2) activity/ expression between others [4]. In patients with corticosteroid resistance, alternative anti-inflammatory treatments are being investigated as well as drugs that may reverse the molecular mechanisms of glucocorticoid resistance. Therefore, the understanding of corticosteroid resistant mechanisms, as biomarkers of therapeutic response, could be of potential value to individualize COPD therapy.

Mucin 1 (MUC1) is a member of membrane-bound mucins that has been associated with airway epithelial anti-inflammatory effects through the inhibition of bacteria and virus activation of toll-like receptors (TLR)-2–9 [5–8]. The MUC1 protein acts as a membrane receptor comprising two polypeptide subunits: an N-terminal extracellular subunit and a C-terminal subunit. A highly conserved cytoplasmic tail (CT) in the C-terminal subunit modulates multiple intracellular signals [9] implicated in different cellular processes such as cellular adherent junction maintenance and morphogenesis [10]. In COPD, MUC1-CT dysregulation promotes the loss of bronchial epithelial barrier integrity [11]. There are several intracellular mediators that can interact with MUC1-CT. Recent data published by our group indicate that MUC1-CT is downregulated in patients with chronic rhinosinusitis with nasal polyps who did not respond to systemic corticosteroids [12]. In vitro studies demonstrated that MUC1-CT forms a complex with GR in response to corticosteroids, mediating the nuclear complex translocation and the induction of anti-inflammatory gene expression. Although MUC1 could be a potential biomarker of corticosteroid response, currently there is no evidence on the association of the lack of MUC1-CT expression and corticosteroid resistance in COPD. The present work analyzes the association of MUC1-CT with corticosteroid efficacy using multiple approaches, including translational, cellular in vitro and animal in vivo models.

## Materials and methods

Unless stated otherwise, all reagents used were obtained from Sigma Chemical Co. (Madrid, Spain).

### Patients

Sputum neutrophils, peripheral blood neutrophils and bronchial epithelial cells were obtained from COPD patients, healthy smokers and healthy non-smoker subjects. Clinical characteristics of patients are defined in Table 1. Twenty-two patients with COPD, defined according to Global Initiative for Chronic Obstructive Lung Disease (GOLD) 2013 guidelines, were enrolled in this study. Patients were aged 67.5 ± 8 years, had forced expiratory volume in 1 s (FEV_1_) of 48.6 ± 8% predicted, and all were prescribed an inhaled corticosteroid, long-acting anticholinergic and long-acting beta 2 agonists. All COPD patients were current smokers and bronchitic. The minimum washout period in stable COPD patients for sampling sputum or blood was 4 days, which avoided effects of chronic medication on the results.COPD patients in this study were classified as GOLD 2 (moderate disease; *n* = 3) and GOLD 3 (severe disease; *n* = 19). There were no exacerbations of the disease within 2 weeks prior to taking blood samples. Fifteen age-matched non-smoking control healthy subjects with normal lung function (age 66 ± 4 years old, FEV1 98 ± 3% predicted) and fifteen healthy smokers (age 65 ± 9 years old, FEV1 89 ± 6% predicted) were also recruited. Routine lung function tests were performed to evaluate forced vital capacity (FVC), FEV1 and FEV1/FVC ratio using a Vitalograph® alpha III spirometer (Vitalograph, Maids Moreton, UK).

**Table 1:** Clinical features

	Healthy (*n* = 15)	Smokers (*n* = 15)	COPD (*n* = 22)
Age, yr. (mean (SD)	66 ± 4	65 ± 9	67.5 ± 8
Sex (M/F)	11/4	12/3	17/5
Tobacco consumption, pack-yr	0	24.1 ± 6	30.5 ± 8
FEV1, % pred	98 ± 3	89 ± 6	48.6 ± 8*
FVC, % pred	91 ± 2	90 ± 4	91.5 ± 6
FEV1/FVC %	94 ± 5	88 ± 1	61.2 ± 10*
GOLD 1 (mild) patients, no.	0	0	0
GOLD 2 (moderate) patients, no.	0	0	3
GOLD 3 (severe) patients, no.	0	0	19
GOLD 4 (very severe) patients, no.	0	0	0
Receiving inhaled steroids, no.	0	0	22
Receiving theophyllines, no.	0	0	0
Receiving long-acting b2-agonist, no.	0	0	22
Receiving anticholinergics, no.	0	0	22
Total peripheral blood neutrophils	4.2 ± 0.6 × 10^9^/L	6.5 ± 0.9 × 10^9^/L*	9.5 ± 2.1 × 10^9^/L*

*COPD* Chronic obstructive pulmonary disease, *FEV1* Forced expiratory volume in 1 s, *FVC* Forced vital capacity; Pack-yr = 1 year smoking 20 cigarettes-day. % pred, % predicted. Data are mean ± SDs. * *P* < 0.05 related to Healthy subjects

Peripheral human lung tissue was obtained from 3 types of subjects (Thoracic Surgery and Pathology Services of the University General Consortium Hospital and University and Polytechnic Hospital La Fe, Spain): A) Patients with COPD who were underwent surgery for organ transplantation program (*n* = 15). B) Lung explants of non-smoker healthy donor subjects from the transplant program (were used as controls), without any lung disease (*n* = 11). C) Smoker donor subjects with normal lung function and lung cancer who were undergoing lung surgery for treatment purposes (lung tissue used in this work was as far as possible of the solitary cancer lesion; *n* = 12). Inclusion criteria comprised either non-smokers or current smokers with or without COPD who were free of symptoms of upper respiratory tract infection. After selection based on lung function, all lung tissue samples chosen for the study were checked histologically using the following exclusion criteria: (1) presence of tumor, (2) presence of post stenotic pneumonia, and (3) fibrosis of lung parenchyma. This project was approved by the local ethics committee of General University Hospital, Valencia, Spain, and written informed consent was taken from each patient or volunteer before starting blood sampling and lung function testing.

### Isolation and culture of primary bronchial epithelial cells

Human bronchial epithelial cells (HBECs) from small bronchi were isolated as previously outlined [13]. Small pieces of human bronchi (0.5–1 mm internal diameter) were excised from microscopically normal lung areas, carefully dissected from lung parenchyma and plated on collagen-coated culture dishes (10 μg/cm^2^ rat type I collagen; Sigma) in bronchial epithelial growth medium (BEGM), comprising bronchial epithelial basal medium (BEBM) supplemented with bovine pituitary extract (52 μg/ml, hydrocortisone 0.5 μg/ml), human recombinant epidermal growth factor ([EGF] 25 ng/ml), epinephrine (0.5 μg/ml), transferrin (10 μg/ml), insulin (5 μg/ml), retinoic acid (50 nM), triiodo-L-thyronine (6.5 ng/ml), gentamycin (40 μg/ml), amphotericin B (50 ng/ml), and bovine serum albumin (1.5 μg/ml). Small bronchi were oriented with the epithelial layer in contact with the culture plate. After a period of ~ 1–2 weeks, bronchial epithelial cells were observed around the bronchi. After trypsinization (passage 2–4), cells were cultured accordingly for different experiments. All the experiments performed in this study with primary HBEC were done on monolayer cultures. The identity of the monolayer as bronchial epithelial cells was confirmed using morphological criteria and immunofluorescence for cytokeratin 5 (KRT5) as well as later use of in vitro differentiation in air-liquid interface as pseudo-stratified bronchial epithelium with basal cells, ciliated cells, columnar, and goblet cells (data not shown). Cell viability was assessed by vital trypan blue exclusion analysis using the Countness® automated cell counter (Life Technologies, Madrid, Spain). Cell viability was > 98% in all cell cultures.

The bronchial epithelial BEAS2B cell line was obtained from American Type Culture Collection and cultured in BEGM media with supplements (Lonza, Madrid, Spain) on collagen-coated culture dishes (10 μg cm^− 2^; rat type I collagen) at 37 °C with 5% CO_2_ in humidified air. The culture medium was replaced every 48 h.

### Human neutrophil isolation from sputum and peripheral blood

Neutrophils were isolated from peripheral venous blood and cultured as previously outlined [14]. using 3% dextran 500 (in 0.9% saline) together with Ficoll-Paque Histopaque 1077 (Amersham Pharmacia Biotech, Barcelona, Spain) at a ratio of 2:1. The neutrophil preparations were > 97% pure as assessed by Giemsa staining and had viability of > 99% as measured by trypan blue exclusion. Neutrophils from spontaneous sputum (~ 2 ml) were collected and processed with dithiothreitol using established methods [15]. Sputum cell pellets were resuspended in RPMI 1640 supplemented with 10% foetal calf serum, 1% penicillin–streptomycin and 1 mmol l-glutamine/L at a concentration of 1 × 10^6^ cells/ml. An aliquot containing 4 × 10^5^ cells was incubated on a 24-well plate for 1 h at 37 °C in humidified 5% CO_2_. Preparations containing < 95% neutrophils were discarded. Neither the purity nor the viability of the cell preparations was affected by the different experimental conditions of the study.

### Preparation of cigarette smoke extract solutions

Cigarette smoke extract (CSE) was prepared as previously outlined [16]. Briefly, the smoke of a research cigarette (2R4F; Tobacco Health Research, University of Kentucky, KY, USA) was generated by a respiratory pump (Apparatus Rodent Respirator 680; Harvard, Germany) through a puffing mechanism similar to the human smoking pattern (3 puffs/min; 1 puff volume of 35 ml; each puff duration lasting 2 s with 0.5 cm above the filter) and was bubbled into a flask containing 25 ml of pre-warmed (37 °C) Roswell Park Memorial Institute (RPMI)-1640 culture medium. The CSE solution was sterilized by filtration through a 0.22 μm cellulose acetate sterilizing system (Corning, USA). The resulting CSE solution was considered 100% CSE and was used within 30 min of preparation. CSE 10% approximately corresponds to the exposure associated with smoking of two packs per day [17]. The quality of the prepared CSE solution was assessed based on the absorbance at 320 nm, which is the specific light absorption wavelength of peroxynitrite. Stock solutions with an absorbance value of 3.0 ± 0.1 were used. To test for cytotoxicity and apoptosis due to CSE, bronchial epithelial cells were treated with CSE concentrations of up to 10% for 24 h. No significant differences in the lactate dehydrogenase supernatant level (lactate dehydrogenase cytotoxicity assay; Cayman, Spain) or in the number of apoptotic cells (annexin V-FITC) were observed in comparison with the control group [18].

### Histology and immunofluorescence analysis

Lung histology was conducted as previously reported in the smoking animal model [19]. Tissue blocks (4 μm thickness) were stained with haematoxylin-eosin for assessment of the inflammatory lung infiltration. Immunofluorescence of MUC1-CT and GRα were analyzed in bronchial epithelial cell cultures and in bronchial epithelium from animals.

The in vitro cell culture of human bronchial epithelial cells was fixed (4% paraformaldehyde, 30 min, at room temperature) after appropriate treatments. Cells were permeabilized (20 mM HEPES at pH 7.6, 300 mM sucrose, 50 mM NaCl, 3 mM MgCl_2_, 0.5% Triton X-100), blocked (10% goat serum in PBS), and incubated with the primary antibodies MUC1-CT (rabbit anti-human polyclonal MUC1-CT antibody (Novus Biologicals (NBP1–60046)) and GRα (mouse anti-human monoclonal glucocorticoid receptor α antibody (GRα; BD Biosciences (611227)) overnight at 4 °C, followed by secondary antibody anti-rabbit/mouse rhodamine/FITC- (1:100, Molecular Probes). Cell nuclei were marked with 49–6-diaidino-2-phenylindole dihydrochloride (1:10,000; DAPI).

Mouse lung tissue was fixed in paraformaldehyde (4%) for 48 h and embedded in Tissue-Tek® OCT™ cryosectioning compound (Sakura Finetek Europe BV, Leiden). Blocks were cut into 10 μm thick sections, permeabilized in Triton X 100 (0.1% in PBS) for 5 min, blocked in 10% goat serum in PBS and co-immunostained with MUC1-CT and GRα antibodies for 24 h at 4 °C followed by a secondary FITC or rhodamine conjugated anti-mouse/rabbit IgG antibody and finally DAPI (2 μg/ml) to mark nuclei (Molecular Probes, Leiden, The Netherlands).

Colocalization of MUC1-CT and GRα was performed using a confocal spectral Leica TCS SP2 microscope with × 1000 magnification and 3× zoom. Red (HeNe 543 nm), green (HeNe 488 nm), and blue (Ar 351 nm, 364 nm) lasers were used. Colocalization studies were performed using the Leica confocal software v2.61.

### Cell stimulations and IL-8, GM-CSF, MIP3a and MMP-9 assays

Primary HBECs or isolated human neutrophils from non-smoker, smoker, and smokers with COPD were adjusted to 500 × 10^6^ cells per well in 6-well plates and incubated in BEGM culture medium at 37 °C with 5% CO_2_. Cells were then treated in the presence or absence of dexamethasone (0.1 nM-1 μM; Sigma Aldrich, Madrid, Spain), for 1 h. After drug incubation, HBECs or neutrophils were stimulated with the TLR4 agonist lipopolysaccharide (LPS) (Invivogene, Toulouse, France) at 1 μg/ml final concentration alone or in combination with CSE at the indicated times as previously outlined [12, 20].

In additional experiments, scrambled siRNA control or MUC1 gene-targeted siRNA were transfected to Beas2B cells for 48 h. Cells were then incubated with dexamethasone 1 μM for 1 h and stimulated with/ without CSE.

Supernatants were collected and centrifuged at 120 g for 5 min. IL-8, GM-CSF, MIP3α or MMP9 were measured in cell-free supernatants using commercially available enzyme-linked immunosorbent assay kits (R&D Systems, Nottingham, UK).

### Real-time RT-PCR and siRNA experiments

Total RNA was isolated from HBECs, human neutrophils, human and animal lung tissue using TriPure® Isolation Reagent (Roche, Indianapolis, USA). The integrity of the extracted RNA was confirmed with Bioanalyzer (Agilent, Palo Alto, CA, USA). Reverse transcription was performed in 300 ng of total RNA with a TaqMan reverse transcription reagents kit (Applied Biosystems, Perkin-Elmer Corporation, CA, USA). cDNA was amplified with specific primers and probes predesigned by Applied Biosystems for TLR4 (Hs00152939_m1), MUC1 (Hs00159357_m1) and MKP1 (Hs00610256_g1) for human samples, and MUC1 (Mm00449604_m1), GRα (Mm00449604_m1), MKP1 (Mm00457274_g1), CD200 (Mm00487740_m1), RGS2 (Mm00501385_m1), TSC22D3 (Mm00726417_m1), IL-8 (Mm04208136_m1), IL-13 (Mm99999190_m1) and CRISPLD (Mm01240812_m1) for mouse samples in a 7900HT Fast Real-Time PCR System (Applied Biosystems) using Universal Master Mix (Applied Biosystems).

MKP1 is a mitogen-activated protein kinase phosphatase whose expression is increased by corticosteroids and induces the degradation/ de-phosphorylation of several kinases such as ERK1/2 and p38, thus increasing the anti-inflammatory properties of corticosteroids [21].

RGS2 is a GTPase-activating protein that switches off signaling from Gq-linked G protein-coupled receptors (GPCRs). In airway smooth muscle, RGS2 is upregulated in a positive cooperative manner by glucocorticoid and LABA, and exerts a bronchoprotective effect; in epithelial and other cells RGS2 expression may attenuate proinflammatory mediator release [22].

TSC22D3 is a glucocorticoid-inducible gene that inhibits the transcriptional activity of both NF-kB and AP-1. Glucocorticoid-induced TSC22D3 expression may also be modestly enhanced by LABAs. It is reported that induction of this gene suppresses various indices of inflammation [23].

Previously known to mediate several other functions, CRISPLD2 was recently found to encode as a novel, secreted, mammalian LPS binding protein in both humans and mice. Enhancement of fluticasone propionate induced CRISPLD2 expression could contribute to the reduction in exacerbations in COPD produced by infections with gram-negative bacteria by downregulating TLR4-mediated proinflammatory responses [24].

CD200 is a glucocorticoid-inducible gene. Pulmonary alveolar macrophages have high constitutive expression of CD200R. Signaling through this receptor involves the interaction of CD200R-bearing cells with other cells (e.g., airway epithelia) that express CD200. Studies in mice have shown that the CD200/CD200R interaction blunts macrophage activation measured as proinflammatory cytokine generation. Acute exacerbations of COPD are triggered, primarily, by prolonged bouts of excessive inflammation in response to bacterial and viral infections. Pharmacological upregulation of CD200 on epithelial and other airway cells in COPD could attenuate inflammation and reduce exacerbation frequency [25].

Expression of the target gene was expressed as the fold increase or decrease relative to the expression of human GAPDH (4310884E) or mouse GAPDH (4352339E) as an endogenous control (Applied Biosystems). The mean value of the replicates for each sample was calculated and expressed as the cycle threshold (Ct). The level of gene expression was then calculated as the difference (ΔCt) between the Ct value of the target gene and the Ct value of GAPDH. The fold changes in the target gene mRNA levels were designated 2^-ΔCt^.

Small interfering RNA (siRNA), including the scrambled siRNA control, was purchased from Ambion (Huntingdon, Cambridge, UK). MUC1 gene-targeted siRNA (identification no. 4392420) was designed by Ambion. Beas2b cells were transfected with siRNA (50 nM) in serum and antibiotic-free medium. After 6 h, the medium was aspirated and replaced with medium containing serum for a further 42 h before cell stimulation. The transfection reagent used was lipofectamine-2000 (Invitrogen, Paisley, UK) at a final concentration of 2 μg/ml.

### Western blotting analysis

Western blotting analysis was used to detect changes in lung tissue expression of MUC1-CT, TLR-4, phospho-ERK1/2, MKP1, GRα and phospho-GR-Ser226, phospho-p38. Lung tissue from human and mouse were homogenized and lysed on ice with a lysis buffer comprising a complete inhibitor cocktail plus 1 mM ethylenediaminetetraacectic acid (Roche Diagnostics Ltd., West Sussex, UK) with 20 mM Tris base, 0.9% NaCl, 0.1% Triton X-100, 1 mM dithiothreitol, and 1 mg/mL pepstatin A. The Bio-Rad assay (Bio-Rad Laboratories Ltd., Herts, UK) was used according to the manufacturer’s instructions to quantify the level of protein in each sample to ensure equal protein loading. Sodium dodecyl sulfate polyacrylamide gel electrophoresis was used to separate the proteins according to their molecular weight. Briefly, 15 μg of proteins (denatured) along with a molecular weight protein marker (Bio-Rad Kaleidoscope marker; Bio-Rad Laboratories) were loaded onto an acrylamide gel consisting of a 5% acrylamide stacking gel stacked on top of a 10% acrylamide resolving gel and run through the gel by application of 100 V for 1 h. Proteins were transferred from the gel to a polyvinylidene difluoride membrane using a wet-blotting method. The membrane was blocked with 5% Marvel in PBS containing 0.1% Tween20 (PBS-T), probed with polyclonal MUC1-CT antibody (Novus Biologicals (NBP1–60046)), monoclonal phospho-ERK1/2 (Sigma-Aldrich (M9692)), monoclonal TLR4 antibody (Novus Biologicals (H00007099-M02)), monoclonal glucocorticoid receptor α antibody (GRα; BD Biosciences (611227)), monoclonal phospho-GR-Ser226 antibody (Novus Biologicals (NB100–92540)), polyclonal Mitogen Activated Protein Kinase Phosphatase 1 antibody (MKP1) (Assay Biotech (B1099), and monoclonal phospho-p38 antibody (Cell Signaling (4511)), and normalized to total mouse anti-human β-actin antibody (monoclonal antibody, catalog no. A1978; Sigma). The enhanced chemiluminescence method of protein detection using enhanced chemiluminescence reagents (ECL Plus; Amersham GE Healthcare, Buckinghamshire, UK) was used to detect labeled proteins. Densitometry of films was performed using the Image J 1.42q software (available at http://rsb.info.nih.gov/ij/, USA). Results of target protein expression are expressed as the percentage of the densitometry of the endogenous controls β-actin.

### Immunoprecipitation

Equal amounts of protein (200 μg) were incubated with 2 μg of anti-GRα or anti-MUC1-CT antibodies and the IgG isotype control. The immune complexes were precipitated with protein G on Sepharose 4B fast flow beads (Sigma (P-3296)) overnight at 4 °C. After washing three times with NET buffer containing 50 mM Tris-HCl at pH 8.0, 150 mM NaCl, and 0.1% Nonidet P-40, the bound materials were eluted from the immunoprecipitates in reducing SDS-PAGE loading buffer containing 10% SDS, 1 M Tris-HCl at pH 6.8, 50% glycerol, 10% 2-mercaptoethanol, and 2% bromophenol blue at 100 °C for 10 min. Immunoprecipitated protein complexes were assayed by western blotting as described above and probed using anti-GRα or anti-MUC1-CT antibodies, as appropriate.

### Glucocorticoid response element transfection assay

Beas2B epithelial cells were seeded (40.000 cells/well) and cultured for 24 h under a 5% CO_2_/air atmosphere at 37 °C in 96-well plates containing Dulbecco’s modified Eagle’s Medium (DMEM). The Cignal GRE Reporter Assay Kit (QIAGEN, Cat no. 336841) was used to monitor the activity of glucocorticoid receptor-induced signal transductions pathways in cultured cells, following the manufacturer’s indications. First, cells were transfected with MUC1 gene-targeted siRNA or scrambled siRNA control as described above. 24 h later, cells were transfected with Cignal Reporter (100 ng), Cignal negative control (100 ng) and Cignal positive control (100 ng) in Opti-MEM serum-free culture medium using Lipofectamine 2000 Reagent (Invitrogen) as transfection reagent. Subsequently, cells were incubated with the transfection reagents at 37 °C in a 5% CO2 incubator for 16 h. After this incubation period, cells were pre-incubated during 6 h with different dexamethasone concentrations (0.1 nM-1 μM) in DMEM.

After this 6 h period, the luciferase assay was developed by using Dual-Luciferase Reporter Assay System (Promega, Cat N° 1910) following the manufacturer’s protocol for developing the assay. In brief, growth medium was removed from the cultured cells and the surface of the cultured cells was washed gently with phosphate buffered saline (PBS). After removing completely the rinse solution, Passive Lysis Buffer 1X was added. The culture plate was then placed on an orbital shaker, with gentle shaking at room temperature for 15 min. In the meantime, Luciferase Assay Reagent II (LAR II) was prepared by resuspending the provided lyophilized Luciferase Assay Substrate in 10 mL of the supplied Luciferase Assay Buffer II. A volume of 100 μl of LAR II was predispensed in the appropriate number of wells of a white 96-well plate. Then, a volume of 20 μl of cell lysate was added into the wells containing LAR II, and mixed by pipetting 2 or 3 times. The white plate was placed in the luminometer (Victor), reading was initiated and the Firefly luciferase activity was measured. In the meantime, the Stop & Glo Reagent was prepared, just before use, by diluting 1 volume of the Stop & Glo Substrate to 50 volumes of Stop & Glo Buffer. Following the first measure, 100 μl of Stop & Glo Reagent was dispensed in the corresponding wells. The plate was replaced in the luminometer and a second reading was initiated and the Renilla Luciferase activity was recorded. Data are expressed as 2xGRE fold induction of luciferase relative to unstimulated cells.

### Inflammatory cigarette smoke animal model

Experimentation and handling were performance in accordance with the guidelines of the Committee of Animal Ethics and Well-being of the University of Valencia (Valencia, Spain). The animal studies followed the ARRIVE guidelines [26]. Mice studies used pathogen-free female C57BL/6 of 12 weeks years old (Harlan Iberica®, Barcelona, Spain). Muc1-KO mice C57BL/6 animals were originally generate by Sandra Gendler (Mayo Clinic, NY, USA) [27] and donated by the animalerie Institut de Médecine Prédictive et de Recherche Thérapeutique, (Lille Cedex, France). Mice were housed with free access to water and food under standard conditions: relative humidity 55 ± 10%; temperature 22 ± 3 °C; 15 air cycles/ per hour; 12/12 h Light/Dark cycle.

The experimental procedure started at day 0 with the basal measure of micro-computed tomography (CT) coupled to positron emission tomography (PET) lung image analysis (micro-CT-PET Albira Imaging System (Oncovision®, Spain) and measure of lung function by plethysmography of whole body (Miami, Florida, USA). At day 1 intranasal lipopolysaccharide (LPS from Salmonella Typhimurium, Sigma Aldrich, Madrid, Spain) or vehicle (sterile deionized water) was administered at 75 μg in 25 μl as previously outlined to induce an inflammatory response [28]. Between days 2 to day 3, mice were exposed to cigarette smoke of 6 cigarettes (2R4F; Tobacco Health Research, University of Kentucky, KY, USA). Between day 4 and day 5 mice were exposed to cigarette smoke of 8 cigarettes. The last day of procedure (day 6), mice were exposed to cigarette smoke of 10 cigarettes, and anlaysis of micro-CT-PET and lung function were assessed. Each cigarette exposure was programed to be consumed in 5 min followed by 8 min of airway exposure using a flux of 5 L/min [29]. A nose only system (TSE systems, Bad Homburg, Germany) was employed for cigarette smoking. Each drag had 1.9 s, and the period of time between drags was 20 s. Control animals received same conditions inhaling air instead of cigarette smoke. Dexamethasone was given at 10 mg/kg/day and 3 mg/kg/day in methocel 10% suspensions, once daily, p.o. by gavage in a volume of 10 mL/kg, starting at day 1 until day 6 of the experimental procedure. Dexamethasone was administer 1 h before cigarette smoke exposure. The account for experimental groups was estimated in a number of 8 mice (*n* = 8) based in previous findings [29]. The primary outcome was the 50% reduction of the lung inflammation (SUV) by dexamethasone 3 mg/kg/day. The sample size was calculated for an effect size f of 1.8, α error of 0.05 and power of 0.96, using one tailed analysis of variance (ANOVA).

Different experimental groups were assigned as follow: 1) Intranasal sterile deionized water + airway nose only system exposure (n = 8); 2) Intranasal sterile deionized water + airway nose only system exposure + dexamethasone 10 mg/kg/day (n = 8); 3) Intranasal LPS + airway nose only system exposure (n = 8); 4) Intranasal LPS + cigarette smoke nose only system exposure (n = 8); 5) Intranasal LPS + cigarette smoke nose only system exposure + dexamethasone 10 mg/kg/day (n = 8); 6) Intranasal LPS + cigarette smoke nose only system exposure + dexamethasone 3 mg/kg/day (n = 8); 7) Intranasal LPS + airway nose only system exposure + 10 mg/kg/day (n = 8); 8) Intranasal LPS + airway nose only system exposure + 3 mg/kg/day.

### Micro-CT-PET analysis

The animal images were acquired at day 0 and 6 using the multimodal micro-PET-CT system (micro-CT-PET Albira Imaging System (Oncovision®, Spain). Animals were anesthesized with intraperitoneal mixture of ketamin (70 mg/Kg) and medetomidin (0.25 mg/kg). After 10 min of stabilization animals were injected intraperitoneally with 300 μCi.

fludeoxyglucose ^18^F (^18^F-FDG). After 30 min, the animals were allocated in supine position in a cradle made of plexiglas to acquire micro-CT and PET images.

Acquisitions of both PET and CT were made with an offset of 10 mm in length to visualize the lung region. Acquisition by CT (Carestream Molecular Imaging, Woodbridge, CT) was performed with good acquisition quality, high dose (400 μA) and high voltage (45Kv) with the Step & Shoot acquisition mode and FBP reconstruction algorithm (Filtered Back Projection). In the PET mode, the studies were programmed with a field of vision (Field Of View (FOV)) of 80 mm, an acquisition time of 90 s per projection during 15 min. The images were reconstructed using the OSEM Cross algorithm with a number of iterations of 3 using the standard reconstruction parameters of the Albira Suite 5.0 Reconstructor software. The images were analyzed and quantified using the PMOD analysis software version 3.2 (PMOD Technologies LTD, Zurich, Switzerland). Once acquired, the PET / CT images were reconstructed and merged.

Volumes of interest (VOI) of the lung region were determined for the CT images. The VOI was analyzed for each subject for their different days of acquisition. For PET images, the VOI obtained in the CT image of each subject was taken as a template and it was extrapolated to the PET image and subsequently quantified. The quantification of images provided the number of accounts acquired per pixel unit. This value was relativized by the dose injected and by subject weight, resulting in the standard uptake value (SUV).

### Statistical analysis

Statistical analysis of results was carried out by parametric (animal and cellular studies) or non-parametric (human tissue studies) analysis as appropriate. *P* < 0.05 was considered statistically significant. Non-parametric tests were used to compare results from human samples of control patients, smoker and COPD patients. In this case, data were displayed as medians and interquartile range values. When the comparisons concerned more than two groups, analysis of variance (Kruskal-Wallis test) was first performed. In the case of a global significant difference, between-group comparisons were assessed by the Dunn’s post-hoc test, which generalizes the Bonferroni adjustment procedure. When the comparisons concerned only 2 groups, between-group differences were analyzed by the Mann Whitney test. Results from animal and cellular in vitro mechanistic cell experiments were expressed as mean ± SE of n experiments since normal distribution for each data set was confirmed by histogram analyses and Kolmogorov–Smirnov test. In this case, statistical analysis was carried out by parametric analysis. Two-group comparisons were analysed using the two-tailed Student’s paired t-test for dependent samples, or unpaired t-test for independent samples. Multiple comparisons were analysed by one-way or two-way analysis of variance followed by Bonferroni post hoc test. Variable correlations were implemented using spearman ρ analysis.

## Results

### MUC1 expression is decreased in lung tissue from smokers and COPD patients

**Fig. 1:**
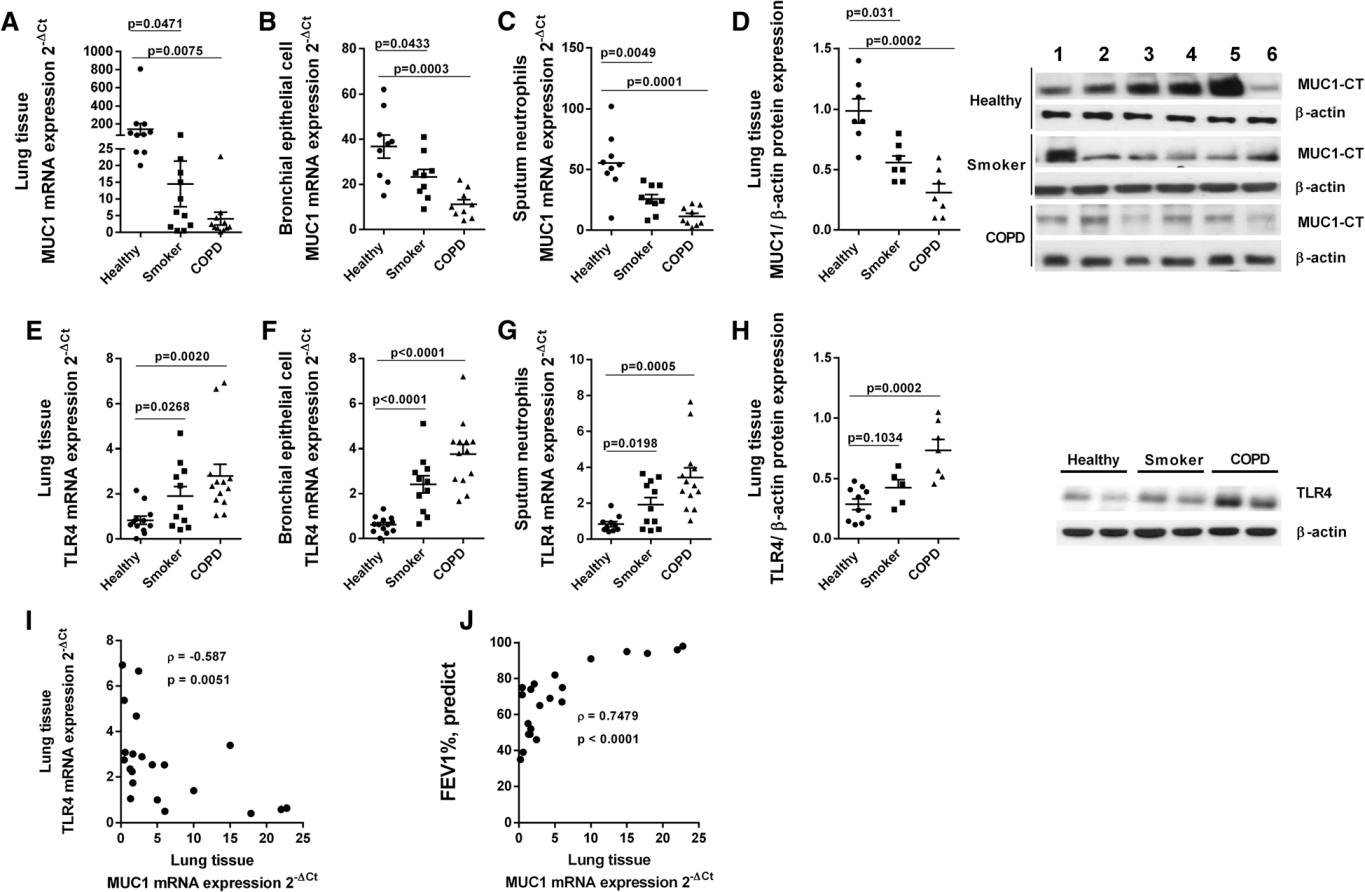
MUC1 downregulation and TLR4 overexpression in lung tissue from heavy smokers and COPD patients. Lung tissue from healthy (*n* = 10), smokers (*n* = 11) and COPD patients (*n* = 13) were analysed. MUC1 mRNA gene expression in lung tissue homogenates (**a**), bronchial epithelial cells (**b**) and sputum neutrophils (**c**). **d** MUC1-CT protein expression in lung homogenates.TLR4 mRNA gene expression in lung tissue homogenates (**e**), bronchial epithelial cells (**f**) and sputum neutrophils (**g**). **h** MUC1-CT protein expression in lung homogenates. **i** Correlation of TL4 and MUC1 gene expression in lung tissue from smokers and COPD patients. **j** Correlation of FEV1% and MUC1 gene expression in lung tissue from smokers and COPD patients. Gene expression was analyzed by real time PCR using the 2^-ΔCt^ as described in methods. Protein expression was analyzed by western blot. Representative western blot are showed. Data are presented as median with interquartile range. “P” exact values were obtained following Kruskal Wallis test. FEV1%: forced expiratory volume in 1 s

Lung tissue, bronchial epithelial cells and neutrophils were isolated from healthy, current smokers and COPD patients based in the clinic characteristic described in Table 1. The gene mRNA expression of MUC1 decreased from 143 (2^-ΔCt^) in healthy lung tissue to 15 (2^-ΔCt^) and 4 (2^-ΔCt^) in lug tissue from smokers and COPD patients respectively (Fig.1a). Similar results were observed in bronchial epithelial cells and sputum neutrophils from smokers and COPD patients (Fig. 1b, c). In the same way, MUC1-CT protein expression was down-regulated in lung tissue from smokers and COPD patients to a 65 and 30% of control respectively (Fig. 1d). The MUC1-CT expression showed the same pattern of MUC1-CT distribution in lung tissue from healthy, smoker and COPD patients (data not shown), mainly located in bronchial epithelial cells and alveolar type II cells as previously described [30]. TLR4 mRNA expression was significantly upregulated in lung tissue, bronchial epithelial cells and sputum neutrophils from smokers and COPD patients (Fig. 1e-g), while TLR4 protein expression only reach significant differences in COPD patients (Fig. 1h). The expression of MUC1 and TLR4 in lung tissue from smokers and COPD patients was inversely correlated (ρ = − 0.587; *P* = 0.0051; Fig. 1i). The FEV1% of smokers and COPD patients was directly correlated with MUC1 lung tissue expression (ρ = − 0.7479; *P* < 0.0001; Fig. 1j).

### Corticosteroid anti-inflammatory effects are impaired in cells from smokers and COPD patients stimulated with cigarette smoke

**Fig. 2:**
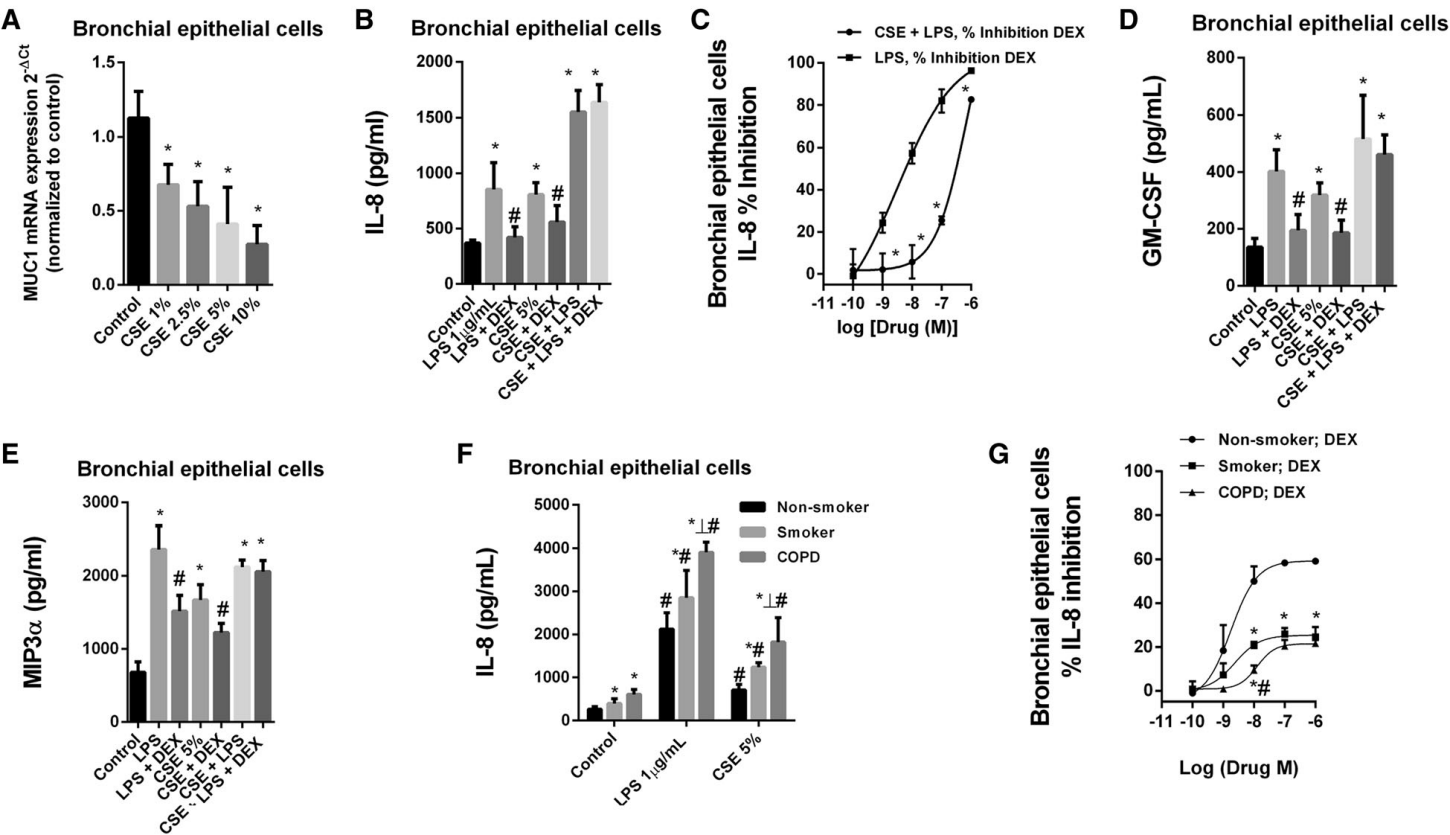
Cigarette smoke mediates corticosteroid resistance and decreases MUC1 expression in human bronchial epithelial cells (HBEC). **a** Cigarette smoke extract (CSE) decreased the mRNA gene expression of MUC1 in HBEC from healthy subjects. **b**-**e** HBECs stimulated with lipopolysaccharide (LPS) and CSE 5% were resistant to the anti-inflammatory effects of dexamethasone on IL-8, GM-CSF and MIP3α release. **f** Both LPS and CSE induce higher IL-8 release in HBECs from smokers and COPD patients than in cells from healthy subjects. **g** Dexamethasone shows lesser inhibitory effect on LPS-induced IL-8 release in cells from smokers and COPD patients than in cells from healthy subjects. The results are shown as mean ± SE of *n* = 4 cell populations from healthy subjects (**a**-**g**), smokers (**f**, **g**) and COPD (**f**, **g**) patients. Gene expression was analyzed by real time PCR using the 2^-ΔCt^ as described in methods. Two-way ANOVA followed by Bonferroni post-hoc tests. **a**, **b**, **d** and **e** *P < 0.05 compared with control; #P < 0.05 compared with stimulus. (**c**) *P < 0.05 compared with CSE+ LPS group. **f** *P < 0.05 compared with non-smoker group; #P < 0.05 compared with control group; ┴ P < 0.05 compared with smoker group. **g** *P < 0.05 compared with non-smoker group; #P < 0.05 compared with smoker group

Cigarette smoke extract (CSE) dose-dependently decreased MUC1 expression in primary bronchial epithelial cells (HBECs) from healthy subjects reaching 73% inhibition at 10% CSE concentration (Fig. 2a). Dexamethasone 1 μM inhibited the LPS-induced IL-8, GM-CSF and MIP3α to 98, 82 and 48% of inhibition respectively (Fig. 2b-d). Similar findings were observed when CSE 5% was used as stimulus (Fig. 2b-c). In contrast, HBECs stimulated with the LPS and CSE combination were resistant to the anti-inflammatory effects of dexamethasone on the IL-8, GM-CSF and MIP3α release (Fig. 2b-e). HBECs from COPD showed higher IL-8 levels than cells from smokers and healthy subjects in response to LPS and CSE (Fig. 2f). Dexamethasone showed more %maximum inhibitory effect (%Emax) on IL-8 release in HBECs from healthy subjects (%Emax 60%) compared with cells from smokers (%Emax 24.5%) and COPD patients (%Emax 21.2%) (Fig. 2g).

**Fig. 3:**
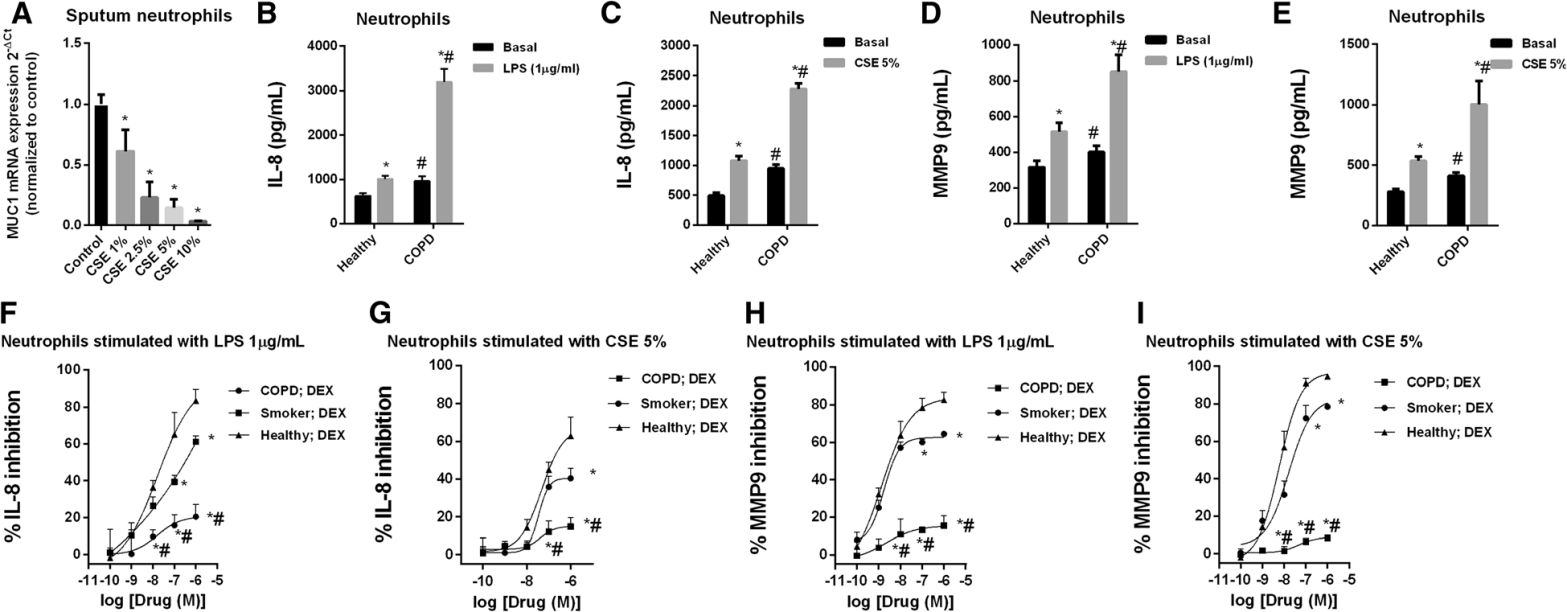
Cigarette smoke mediates corticosteroid resistance and decreases MUC1 expression in human neutrophils. **a** Cigarette smoke extract (CSE) decreased the mRNA gene expression of MUC1 in sputum neutrophils from smokers. **b**-**e** Peripheral blood neutrophils from healthy and COPD patients were stimulated with (**b**, **d**) lipopolysaccharide (LPS) or (**c**, **e**) with cigarette smoke extract (CSE) during 6 h, and IL-8 and MMP9 levels were measured by ELISA. **f**-**i** Peripheral blood neutrophils form healthy, smokers and COPD patients were incubated with dexamethasone for 1 h and stimulated with LPS or CSE for 6 h. The results are shown as mean ± SE of *n* = 4 cell populations from healthy subjects, smokers and COPD patients. Gene expression was analyzed by real time PCR using the 2^-ΔCt^ as described in methods. Two-way ANOVA followed by Bonferroni post-hoc tests. **a**-**e** *P < 0.05 compared with control/basal; #*P* < 0.05 compared with healthy group. **f**-**i** *P < 0.05 compared with healthy group; #*P* < 0.05 compared with smoker group

CSE dose-dependently decreased MUC1 expression in sputum neutrophils, reaching 96% of inhibition after CSE 10% exposure (Fig. 3a). Similar results were observed in neutrophils from peripheral blood (data not shown). LPS and CSE increased IL-8 and MMP9 release in cultured peripheral blood neutrophils which was significantly higher in neutrophils from COPD patients (Fig. 3b-d). Dexamethasone showed impaired anti-inflammatory effects on the LPS and CSE-induced IL-8 and MMP9 release, in neutrophils from smokers and COPD patients (Fig. 3f-i).

### MUC1 mediates corticosteroid anti-inflammatory effects in vitro

**Fig. 4:**
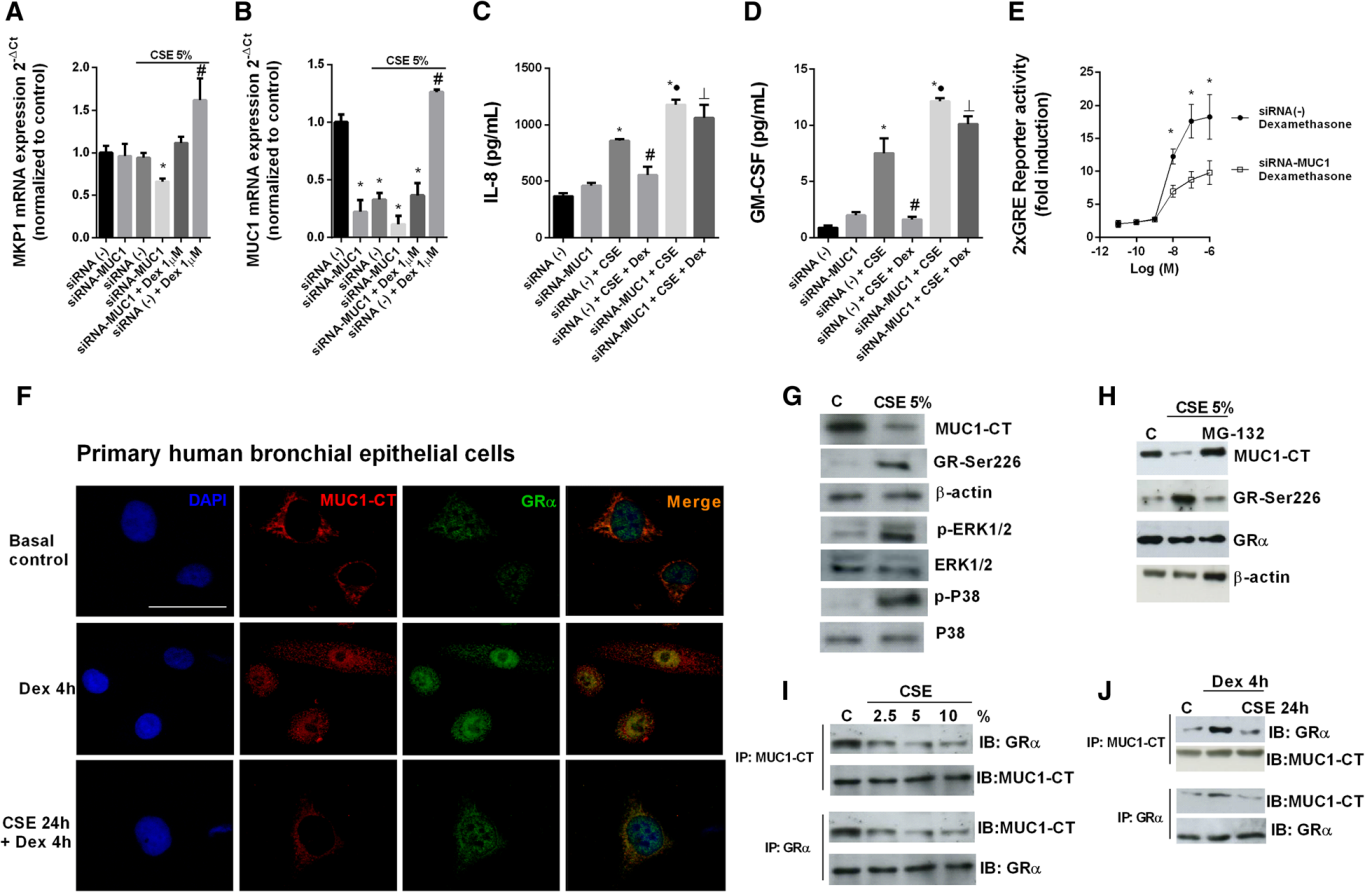
MUC1 mediates dexamethasone anti-inflammatory effects. **a**-**e** Bronchial epithelial Beas2b cells were transiently transfected with siRNA-MUC1 or with control siRNA(−) for 48 h, incubated with dexamethasone (Dex) for 1 h and (**a**-**d**) stimulated with cigarette smoke extract (CSE) during 24 h. **a**, **b** MKP1 and MUC1 mRNA gene expression was analyzed by real time PCR. **c**, **d** IL-8 and GM-CSF release was measured by ELISA. **e** Glucocorticoid response element (GRE) activation was measured after 4 h of dexamethasone incubation at different concentrations in Beas2b cells transfected with Cignal GRE Reporter Assay. **f** Confocal co-immunofluorescence analysis of glucocorticoid alpha (GRα) and MUC1-CT co-localization in primary human bronchial epithelial cells (HBECs) treated with dexamethasone during 4 h, and in cells incubated with CSE for 24 h followed by the incubation with dexamethasone for 4 h. DAPI was used to mark cell nuclei. Scale bar: 5 μm. **g** Protein expression measured by western blot in HBEC stimulated with CSE during 24 h. **h** HBECs were incubated with MG132 5 μM during 1 h followed by CSE stimulation during 24 h. Western blot of different proteins was analysed. **i** Immunoprecipitation of MUC1-CT and GRα in HBECs stimulated with different concentrations of CSE. **j** Immunoprecipitation of MUC1-CT and GRα in HBECs incubated with CSE during 24 h followed by the stimulation with dexamethasone during 4 h. Representative western blot are showed. The results are shown as mean ± SE of n = 4 independent experiments. Gene expression was analyzed by real time PCR using the 2^-ΔCt^ as described in methods. One-way ANOVA followed by Bonferroni post-hoc tests. **a**, **b** *P < 0.05 compared with siRNA(−) transfected cells; #*P* < 0.05 compared with siRNA-MUC1 + Dex group. **c**, **d**) *P < 0.05 compared with siRNA(−) transfected cells; # and ●*P* < 0.05 compared with siRNA(−) + CSE group; ┴*P* < 0.05 compared with siRNA(−) + CSE + Dex group. **e** *P < 0.05 compared with siRNA-MUC1 + Dex group

Beas2b bronchial epithelial cells transiently transfected with siRNA-MUC1 were resistant to the effects of dexamethasone which did not increase the anti-inflammatory MKP1 and MUC1 expression (Fig. 4a, b). In the same way, in transiently transfected Beas2b cells with siRNA-MUC1, dexamethasone did not reduce IL-8 and GM-CSF release in response to CSE (Fig. 4c, d). Corticosteroids mediate part of its anti-inflammatory effects through transactivation of genes that encode proteins with anti-inflammatory properties, such as MKP1, via direct interaction of the agonist-bound GR to glucocorticoid response elements (GREs). In this work, cells were transfected with GRE reporter construct in siRNA-MUC1 cells and siRNA(−) cells and incubated with dexamethasone 1 μM during 4 h. Treatment of 2xGRE Beas2B reporter cells with dexamethasone (0.1 nM to 1 μM) for 4 h induced GRE dependent transcription in a concentration-dependent manner that was significantly lower in siRNA-MUC1 than in siRNA(−) control cells (Fig. 4e).

Confocal immunofluorescence for GRα and MUC1-CT revealed that both proteins are co-expressed in the cytoplasm of primary HBECs around the nucleus. After dexamethasone addition the GRα-MUC1-CT complex was translocated to the cell nucleus. However, pre-treated cells with CSE 5% for 24 h decreased MUC1-CT expression and blocked GRα-MUC1-CT nucleus translocation (Fig. 4f). HBECs incubated with CSE5% for 24 h showed a decreased expression of MUC1-CT, and an increased expression of GR phosphorylated at serine 226 (GR-Ser226), p-ERK1/2 and p-P38 expression (Fig. 4g). The proteasomal inhibitor MG-132 5 μM inhibited the loss of expression of MUC1-CT induced by CSE5% and reversed the increase of GR-Ser226 (Fig. 4h). Immunoprecipitation experiments revealed that MUCI-CT forms a complex with GRα that is dissociated by CSE in a concentration-dependent manner (Fig. 4i). The incubation of HBECs with CSE 5% during 24 h prevented the formation of GRα-MUC1-CT complex induced by dexamethasone (Fig. 4j).

### MUC1 mediates corticosteroid anti-inflammatory effects in vivo

Dexamethasone administered at 3–10 mg/kg/day orally improved respiratory resistance (enhanced pause (Penh)), decreased inflammatory cells (Additional file 1: Data S1A-D), and IL-8/IL-13 (Additional file 1: Data S2A and S2B) in bronchoalveolar lavage (BAL) fluid of WT control animals treated with LPS and in a lesser extent in LPS + CS treated animals. In Muc1 KO animals, dexamethasone did not improve Penh neither number of inflammatory cells nor IL8/IL13 cytokines in BAL fluid (Additional file 1: Data S1 and S2). Muc1 KO mice showed predominant neutrophil inflammation after LPS or LPS + CS exposure (Additional file 1: Figure S1). Lung hematoxylin & eosin histology showed lesser lung inflammatory cell infiltrates in lung sections from WT animals treated with dexamethasone in both groups: animals exposed to LPS and LPS + CS (Additional file 1: Figure S3). In contrast, dexamethasone did not reduced inflammatory cell infiltrates in Muc1 KO animals (Additional file 1: Figure S3).

**Fig. 5:**
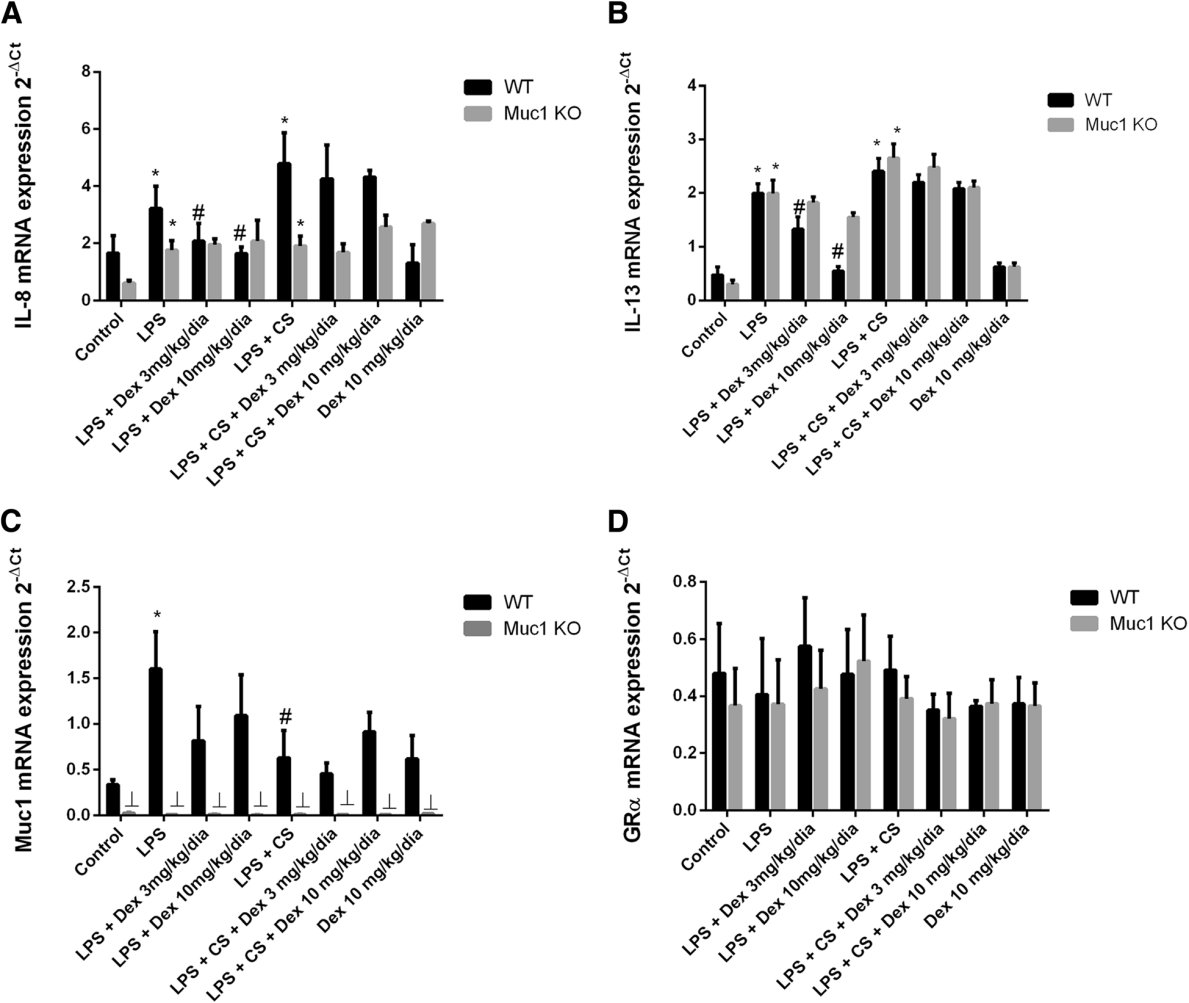
Muc1 KO mice is resistant to the anti-inflammatory effects of dexamethasone in a model of acute cigarette smoke inflammation. C57BL/6 Muc1 KO mice and WT mice were undergoing intranasal instillation of 75 μg of lipopolysaccharide (LPS) at day 1. Between days 2 and 3, animals were exposed to cigarette smoke (or control air) of 6 cigarettes followed by 8 cigarettes (or control air) at days 4 and 5, and 10 cigarettes (or control air) at day 6. Vehicle or dexamethasone at 3 mg/kg/day and 10 mg/kg/day was administered orally once a day between day 1 and 6. Animals were sacrificed at day 6 and lungs were homogenized to analyze the gene mRNA expression of (**a**) IL-8, (**b**) IL-13, (**c**) Muc1 and (**d**) GRα. Gene expression was analyzed by real time PCR using the 2^-ΔCt^ as described in methods. Results are the mean ± SE of *n* = 8 animals per experimental group. One-way ANOVA followed by Bonferroni post-hoc tests. *P < 0.05 compared with control. #*P* < 0.05 compared with LPS. ┴*P* < 0.05 compared with WT. KO: knock out; WT: wild type; CS: cigarette smoke; LPS: lipopolysaccharide

In lung tissue, LPS and LPS + CS administration increased IL-8 and IL-13 mRNA expression (Fig. 5a, b). Dexamethasone inhibited IL-8 and IL-13 expression only in WT animals stimulated with LPS (*P* < 0.05) but not in animals stimulated with LPS + CS (Fig. 5a, b). In Muc1 KO mice dexamethasone did not inhibited IL-8 and IL-13 expression (Fig. 5a, b). Lung tissue from WT control mice increased Muc1 expression in response to dexamethasone (Fig. 5c). Furthermore, LPS stimulus increased the expression of Muc1 in WT animals (Fig. 5c). MUC1-KO mice did not show Muc1 expression in lung tissue confirming the KO nature (Fig. 5c). GRα expression was not affected by experimental conditions (Fig. 5d).

**Fig. 6:**
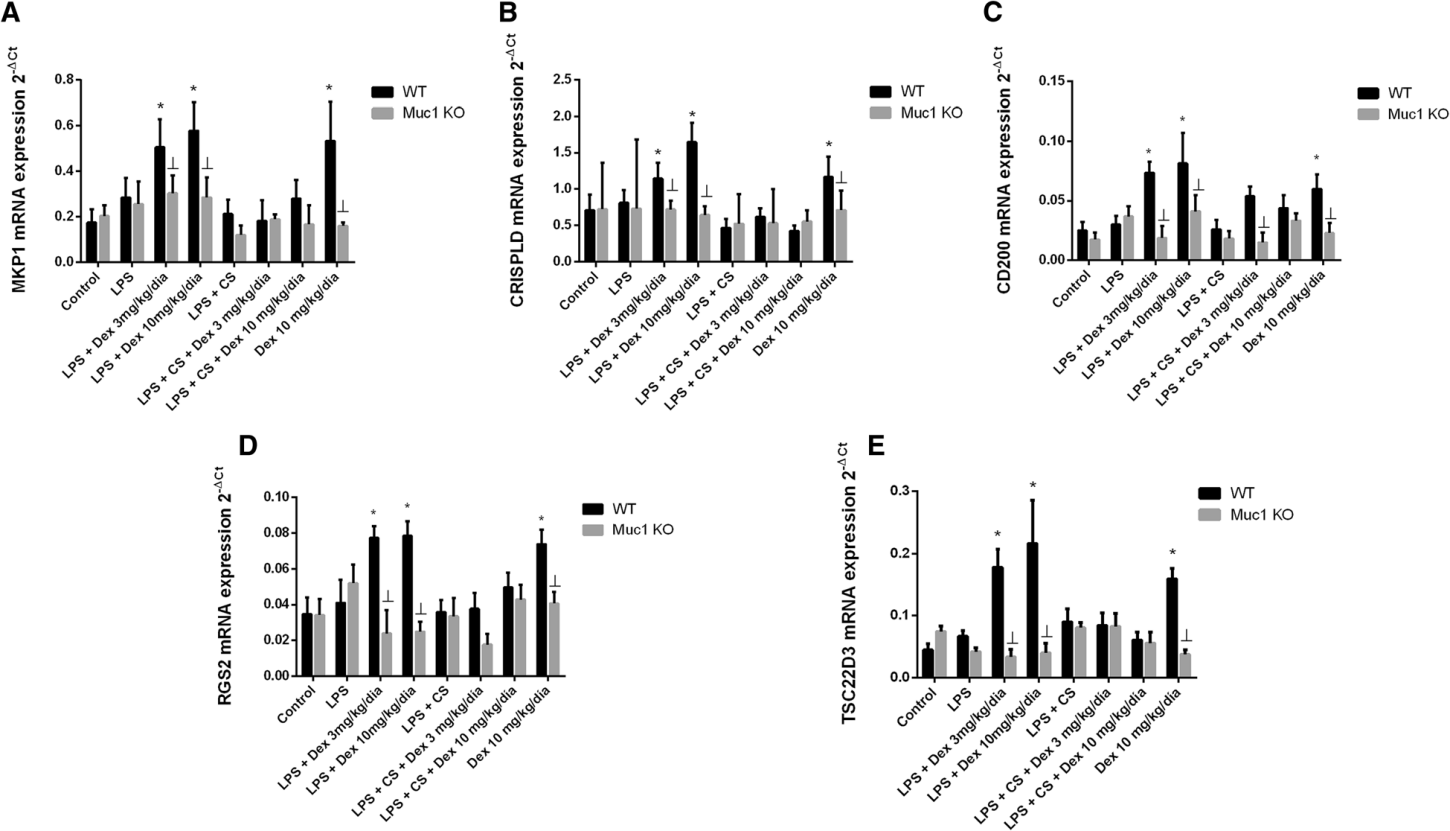
Corticosteroid anti-inflammatory inducible genes expression is resistant to dexamethasone in Muc1 KO mice. C57BL/6 Muc1 KO mice and WT mice were undergoing intranasal instillation of 75 μg of lipopolysaccharide (LPS) at day 1. Between days 2 and 3, animals were exposed to cigarette smoke (or control air) of 6 cigarettes followed by 8 cigarettes (or control air) at days 4 and 5, and 10 cigarettes (or control air) at day 6. Vehicle or dexamethasone at 3 mg/kg/day and 10 mg/kg/day was administered orally once a day between day 1 and 6. Animals were sacrificed at day 6 and lungs were homogenized to analyze the gene mRNA expression of different corticosteroid-inducible genes such as (**a**) MKP1, (**b**) CRISPLD, (**c**) CD200, (**d**) RGS2 and (**e**) TSC22D3. Gene expression was analyzed by real time PCR using the 2^-ΔCt^ as described in methods. Results are the mean ± SE of n = 8 animals per experimental group. One-way ANOVA followed by Bonferroni post-hoc tests. *P < 0.05 compared with control. ┴*P* < 0.05 compared with WT. KO: knock out; WT: wild type; CS: cigarette smoke; LPS: lipopolysaccharide

Corticosteroids can enhance genes that encode proteins with anti-inflammatory properties. In this work, dexamethasone elevated the expression of corticosteroid-inducible anti-inflammatory genes MKP1, CRISPLD, CD200, RGS2 and TSC22D3 in lung tissue of WT control animals stimulated with LPS, but not in animals stimulated with LPS + CS (Fig 6a-e). Furthermore, dexamethasone did not increase the expression of the anti-inflammatory genes in Muc1 KO mice (Fig. 6a-e).

**Fig. 7:**
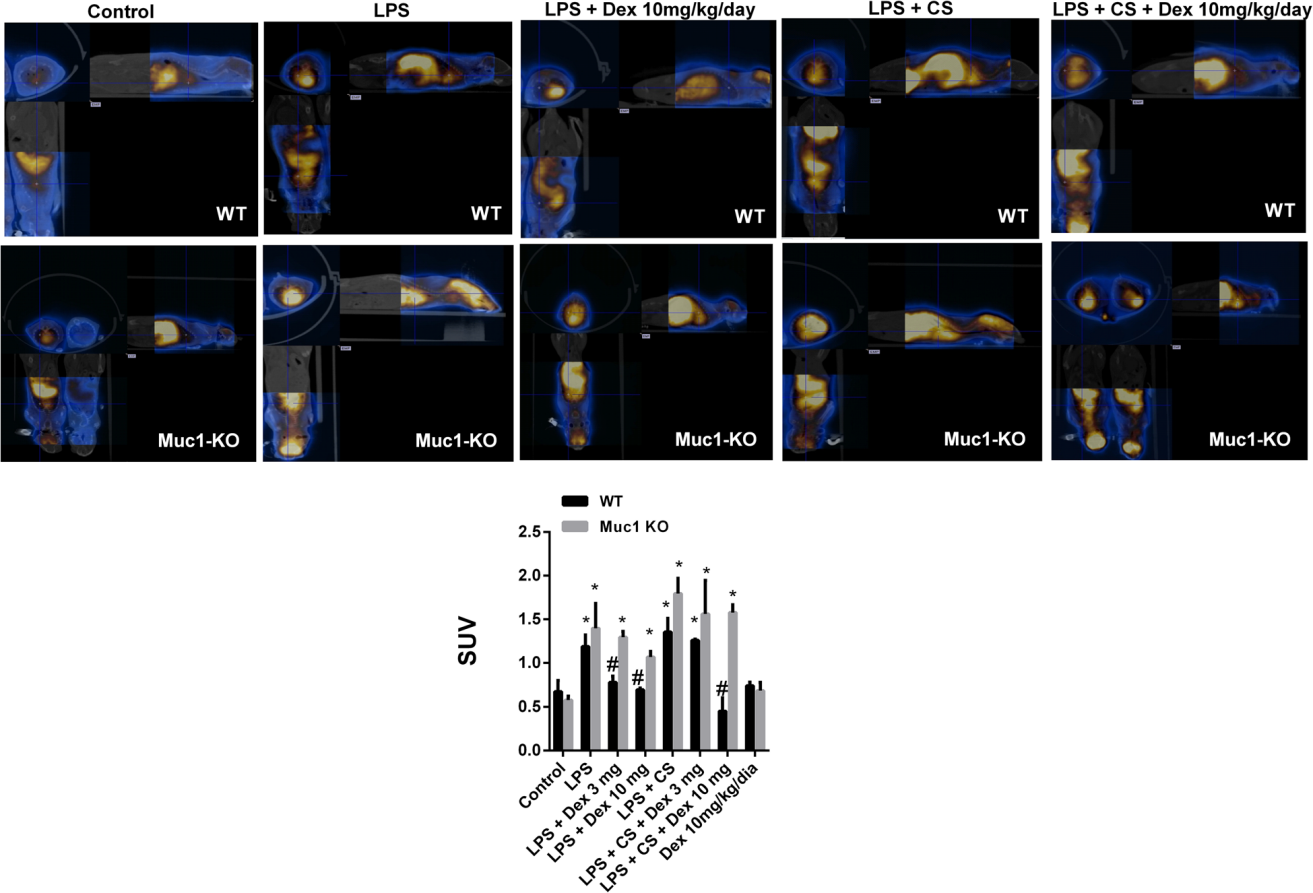
Muc1 KO mice is resistant to the anti-inflammatory effects of dexamethasone in a model of acute cigarette smoke inflammation analysed by micro-CT-PET. C57BL/6 Muc1 KO mice and WT mice were undergoing intranasal instillation of 75 μg of lipopolysaccharide (LPS) at day 1. Between days 2 and 3, animals were exposed to cigarette smoke (or control air) of 6 cigarettes followed by 8 cigarettes (or control air) at days 4 and 5, and 10 cigarettes (or control air) at day 6. Vehicle or dexamethasone at 3 mg/kg/day and 10 mg/kg/day was administered orally once a day between day 1 and 6. Computed tomography (CT) followed by positron emission tomography (PET) of fludeoxyglucose ^18^F (^18^F-FDG) was measured as standard uptake value (SUV). Representative images are showed. Results are the mean ± SE of n = 8 animals per experimental group. One-way ANOVA followed by Bonferroni post-hoc tests. *P < 0.05 compared with control. #*P* < 0.05 compared with LPS/ LPS + CS. KO: knock out; WT: wild type; CS: cigarette smoke; LPS: lipopolysaccharide

Micro-CT-PET image analysis detected an increase of fludeoxyglucose ^18^F (^18^F-FDG) signal expressed as standard uptake value (SUV) in animals treated with LPS and LPS + CS (Fig. 7). Dexamethasone decrease SUV values at 3 mg and 10 mg/kg/day in WT animals stimulated with LPS, while only dexamethasone 10 mg/kg/day was effective inhibiting SUV in animals treated with LPS + CS. In Muc1-KO animals, dexamethasone did not reduce SUV values (Fig. 7).

**Fig. 8:**
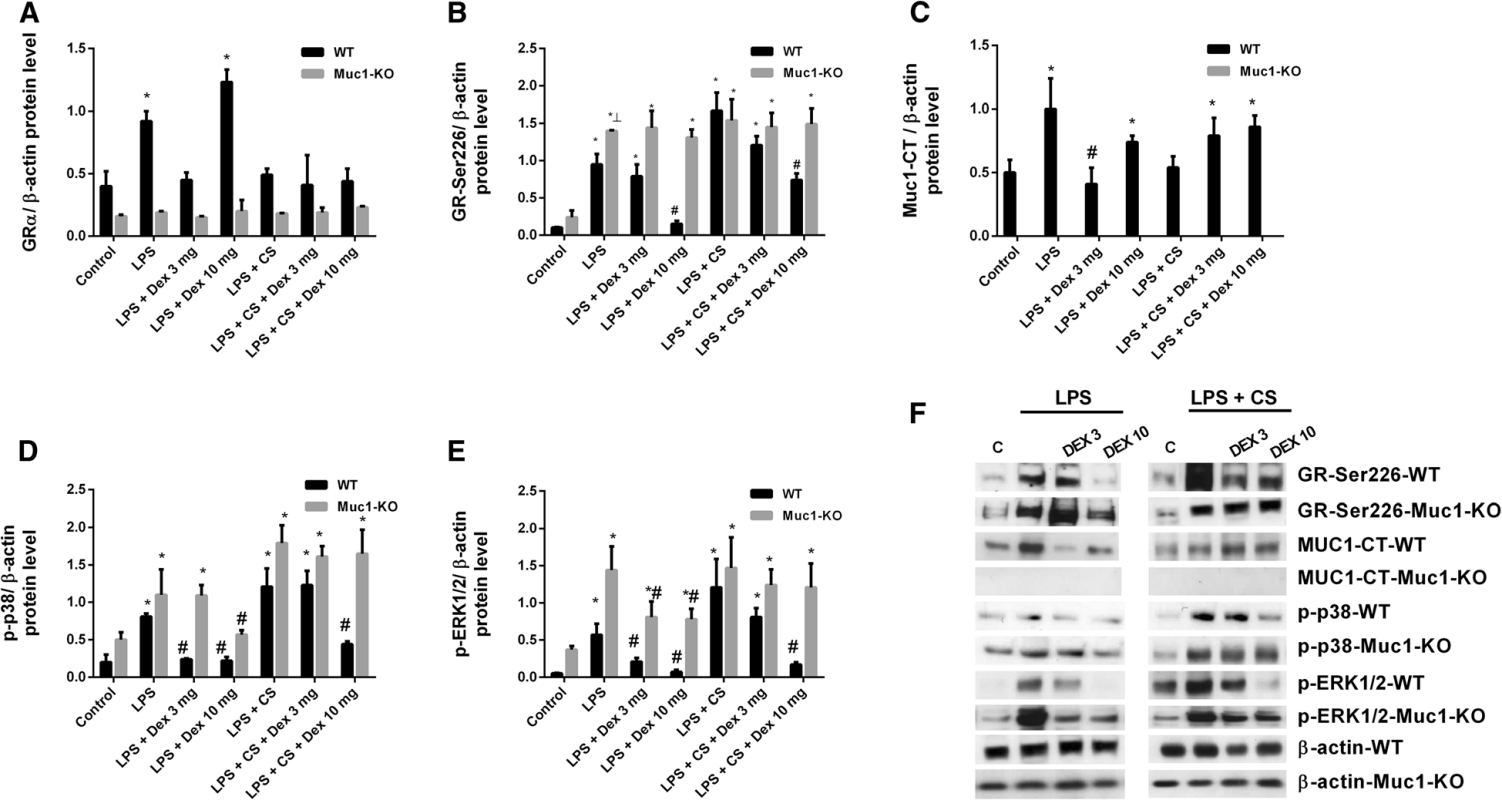
Mechanistic pathways involved in corticosteroid resistance of Muc1 KO mice. C57BL/6 Muc1 KO mice and WT mice were undergoing intranasal instillation of 75 μg of lipopolysaccharide (LPS) at day 1. Between days 2 and 3, animals were exposed to cigarette smoke (or control air) of 6 cigarettes followed by 8 cigarettes (or control air) at days 4 and 5, and 10 cigarettes (or control air) at day 6. Vehicle or dexamethasone at 3 mg/kg/day and 10 mg/kg/day was administered orally once a day between day 1 and 6. Animals were sacrificed at day 6 and lungs were homogenized to analyze protein expression of (**a**) glucocorticoid receptor alpha (GRα), (**b**) GR phosphorylated at ser226 (GR-ser^226^), (**c**) Muc1 cytoplasmic tail (CT), (**d**) phospho-p38 and (**e**) phopho-ERK1/2. Protein expression was normalized to β-actin. **f** Representative western blots are showed. Results are the mean ± SE of n = 8 animals per experimental group. One-way ANOVA followed by Bonferroni post-hoc tests. *P < 0.05 compared with control. #*P* < 0.05 compared with LPS/ LPS + CS. KO: knock out; WT: wild type; CS: cigarette smoke; LPS: lipopolysaccharide

We observed that both LPS and LPS + CS can enhance the GR phosphorylation at ser^226^, while dexamethasone reduces GR-ser^226^ expression in lung tissue of WT control mice (Fig. 8a, b). However, dexamethasone showed impaired effects reducing GR-ser^226^ expression in Muc1 KO animals. LPS inflammation, but not LPS + CS, increased the expression of Muc1-CT in lung tissue from WT control animals (Fig. 8c). Neither LPS nor LPS + CS modified the Muc1-CT distribution (data not shown). Furthermore, dexamethasone at the highest dose, increased the Muc1-CT expression (Fig. 8c). The anti-inflammatory effects of corticosteroids are partially mediated by the inhibition of the phosphorylation of different intracellular pathways. Thus, for example, p38 and ERK1/2 phosphorylations are typically enhanced by LPS and in cells from COPD patients. In this work, dexamethasone reduced the phosphorylation of p38 and ERK1/2 at 3 mg and 10 mg/kg/day in WT controls stimulated with LPS, but only at 10 mg/kg/day when it was stimulated with LPS + CS. In Muc1 KO animals, dexamethasone showed lesser effect inhibiting p38 and ERK1/2 phosphorylation (Fig. 8d-f).

**Fig. 9:**
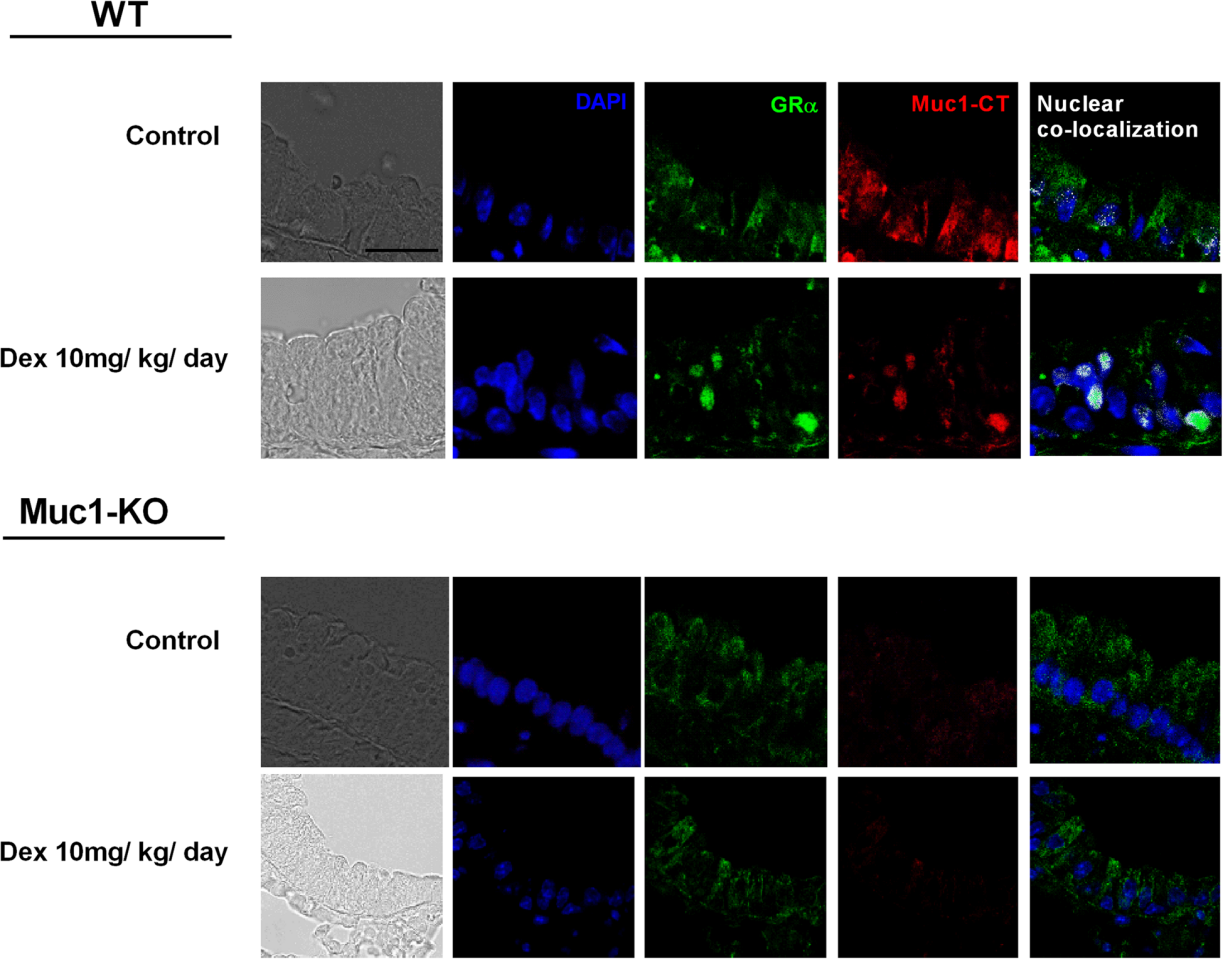
Dexamethasone induces Muc1-CT nuclear translocation and co-localization with GRα in bronchial epithelium of WT mice but not in MUC1 KO mice. C57BL/6 Muc1 KO mice and WT mice were undergoing dexamethasone 10 mg/kg/day (orally) for 6 days. Animals were sacrificed at day 6 and lungs were fixed in paraformaldehyde (4%) for 48 h and embedded in Tissue-Tek® OCT™ cryosectioning compound. Lung slices were immunostained with MUC1-cytoplasmic tail (CT) and glucocorticoid receptor alpha (GRα) antibodies with rhodamine/fluorescein secondary antibodies. DAPI was used to mark cell nucleus. Representative con-focal images are showed. Scale bar: 5 μm

Confocal immunofluorescence showed a Muc1-CT-GRα co-localization in the bronchial epithelial cell cytoplasm of WT mice. The treatment with dexamethasone induced the bronchial epithelial cell nuclear translocation of the Muc1-CT-GRα complex (Fig. 9). In Muc1 KO animals GRα was expressed in bronchial epithelial cell cytoplasm, but nuclear translocation in response to dexamethasone was not observed (Fig. 9).

## Discussion

The present work shows that the loss of MUC1-CT expression mediates corticosteroid resistance in in vitro and in vivo models relevant to the COPD pathology. MUC1-CT was downregulated in lung tissue, isolated bronchial epithelial cells and neutrophils from heavy smokers and COPD patients resistant to corticosteroids. Furthermore, cigarette smoke extract decreased MUC1-CT expression. In vitro cell MUC1 gene silencing and animal in vivo MUC1 KO model of COPD inflammation demonstrated that corticosteroids mediate part of their anti-inflammatory effects by forming MUC1-CT-GRα complexes that translocate to the cell nucleus to increase the expression of anti-inflammatory genes. These results support previous findings in corticosteroid resistant chronic rhinosinusitis with nasal polyps (CRSwNP) [12] which may be of potential value to explain, almost in part, the loss of corticosteroid efficacy in COPD.

The use of ICS in COPD is currently under debate. Recent clinical trials using fixed-dose combinations of LAMA/LABA have demonstrated more effectiveness than the LAMA or LABA alone in terms of improvement in trough FEV1, transitional dyspnea index (TDI) and St. George’s Respiratory Questionnaire (SGRQ) scores [31]. The recent study FLAME showed that the combination of indacaterol (110 μg) plus glycopyrronium (50 μg) was superior to salmeterol (50 μg) plus fluticasone (500 μg) improving FEV_1_ and preventing acute exacerbations of COPD in both moderate and severe COPD patients [32, 33]. However the LABA/ICS combination or the triple therapy LABA/LAMA/ICS seems superior in the cases of very severe airflow limitation [32]. The evidence that dual bronchodilation can prevent or at least delay the onset of acute exacerbations of COPD raises the fundamental questions whether it makes sense to switch all patients from a LABA/ICS regimen to a LABA/LAMA regimen on the basis of the improvement in lung function and the lower exacerbation rates [34] or there is a subgroup of patients with COPD who may benefit the most from this therapy. Perhaps the nature of COPD exacerbations (viral or bacterial) or the inflammatory phenotype (eosinophils vs neutrophils) could help us to appropriately select the correct regimen therapy. In this regard, the role of elevated blood eosinophils as a biomarker for the identification of candidates for ICS treatment is currently debated, although recent pooled analysis have ruled out the association of blood eosinophils as predictor of ICS good response [35].

Therefore, there is a need to understand the different cellular and molecular mechanisms implicated in the loss of corticosteroid efficacy in COPD. A good understanding of corticosteroid resistance could be of potential value to design new strategies such as the addition of novel drugs to reverse corticosteroid resistance [36] or the analysis of corticosteroid resistance biomarkers to avoid ICS therapy. Between the different molecular processes associated to the loss of corticosteroid efficacy, the most analyzed process involves the activation of phosphatidylinositide 3-kinase (PI3K) δ that switch off histone deacetylase 2 (HDAC2) to avoid the correct GRα-corticosteroid inhibition of NF-kB–dependent inflammatory gene expression [37]. Also, GRα modifications such as the phosphorylation at specific serine residues of GRα, such as ser^226^, impedes nuclear translocation, leading to steroid resistance [38]. The sustained inflammation in COPD is believed to increase and perpetuate the GR-ser^226^ status [38]. In this regard, the innate immune system through the chronic stimulation of TLRs activates intracellular p38, ERK1/2 and JNK1 intracellular pathways that can phosphorylate GRα at ser^226^ [39, 40]. TLR4 is activated by lipopolysaccharide (LPS) of gram negative bacteria and cigarette smoke, and its expression appears to be chronically elevated in bronchial epithelial cells from stable severe and very severe COPD patients, contributing to airway chronic inflammation [41]. In this work we confirmed this observations, showing an increase of TLR4 expression in bronchial epithelial cells and neutrophils from heavy smokers and COPD patients. Unlike TLR4, the expression of MUC1-CT was decreased in lung tissue, bronchial epithelial cells and neutrophils from heavy smokers and COPD patients. Cytoplasmic MUC1-CT mediates anti-inflammatory effects through the interaction with TLR signal components [8, 42]. Therefore, the higher expression of TLR4 and low expression of MUC1-CT implicates a greater inflammatory response. In this work, both, LPS and CSE promoted a higher inflammatory response in bronchial epithelial cells and neutrophils from heavy smokers and COPD patients. Furthermore, bronchial epithelial cells transiently transfected with siRNA-MUC1 showed higher amounts of IL-8 and GM-CSF than siRNA controls in response to CSE. In this line, Muc1-KO animals showed higher amount of BAL inflammatory cells and inflammation, thus confirming the anti-inflammatory role of MUC1-CT.

In contrast to our findings, recent data indicate that sputum MUC1 is increased in the acute phase of COPD exacerbation and also in the BALF of mice instilled with intranasal LPS [43]. In this work, intranasal instillation of LPS also increased MUC1 expression in lung tissue that was abrogated with the combination of CS. Difference observed could be explained by the nature of COPD patients studied, since we analyzed samples from stable COPD and not from exacerbations that can trigger MUC1 transiently as anti-inflammatory endogenous control to limit excessive inflammation [43].

The lack of MUC1-CT not only promoted a higher inflammation but reduced the anti-inflammatory effect of corticosteroids. As commented above, the over-activation of TLR4 in absence of MUC1-CT could increase the chronic phosphorylation of p38 and ERK1/2 to promote GR-ser^226^ and consequently inducing corticosteroid resistance. In this work, dexamethasone was unable to inhibit p38 and ERK1/2 phosphorylations in lungs from Muc1 KO mice, thus allowing sustained and elevated levels of GR-ser^226^ which has been related with the lack of GRα cell nuclear translocation [12]. These data was corroborated in confocal microscope and immunoprecipitation in vitro studies using bronchial epithelial cells, showing nuclear translocation of MUC1-CT-GRα complex after dexamethasone stimulation that was inhibited by cigarette smoke pre-incubation. Similar results were observed in vivo in mice Muc1 KO bronchial epithelial cells. The interaction of MUC1-CT forming a complex with GRα has been observed previously in beas2b bronchial epithelial cells and nasal polyp tissue confirming the results of this study [12]. However, MUC1-CT can interact with other proteins. This is the case of the estrogen receptor (ER)-α and β-catenin that can form complexes with MUC1-CT to stabilize and protect ERα and β-catenin from their ubiquitination and degradation allowing nuclear translocation of both proteins [11, 44]. These findings are in line with a possible protective role of MUC1-CT on GRα. In this work, the proteasomal inhibitor MG-132 prevented the CSE-induced MUC1-CT decrease, suggesting that CSE mediates MUC1-CT downregulation via proteasomal degradation. In the same way, proteasomal inhibition reduced the GR-ser^226^ expression, suggesting that MUC1-CT could protect GRα against phosphorylation at serine 226.

Corticosteroids mediates part of their anti-inflammatory effects through the activation of GRE gene regions, thus increasing the expression of corticosteroid dependent anti-inflammatory genes. In this work, bronchial epithelial cells transfected with siRNA-MUC1 showed an impaired GRE activation in response to growing concentrations of dexamethasone that was translated in a lack of expression of corticosteroid inducible anti-inflammatory MKP1 gene. Similar findings were observed in Muc1 KO mice, in which corticosteroid-inducible anti-inflammatory genes MKP1, CRISPLD, CD200, RGS2 and TSC22D3 were resistant to dexamethasone. Interestingly, WT control animals stimulated with LPS and CS were also resistant to the effect of dexamethasone increasing anti-inflammatory genes and inhibiting inflammatory cytokines such as IL8 and IL13 which related CS with resistance to corticosteroids as previously outlined [4].

However, although the findings of this study provide a rational explanation for the lack of efficacy of corticosteroids in patients with COPD, other mechanisms may also be implicated [37], thus sharing different mechanisms that can contribute to the final COPD phenotype.

## Conclusions

We have demonstrated a new role for MUC1-CT in the modulation of the anti-inflammatory effects of corticosteroids in COPD. Our findings suggest a possible explanation for why corticosteroids cannot repress inflammation in COPD, thus contributing to the amount of knowledge of corticosteroid resistance processes in COPD which may be of potential value to develop new strategies to the COPD treatment.

## Additional file

Additional file 1: **Figure S1.** Acute cigarette smoke/ lipopolysaccharide lung inflammatory animal model showed resistance to dexamethasone improving lung resistance and bronchoalveolar inflammatory cell extravasation in Muc1 KO animals. **Figure S2.** IL-8 and IL-13 bronchoalveolar fluid content in Muc1 KO mice exposed to acute cigarette smoke/ lipopolysaccharide is resistant to dexamethasone. **Figure S3.** Inflammatory lung cell infiltration secondary to acute lipopolysaccharide/ cigarette smoke exposure is resistant to dexamethasone in MUC1 KO mice. (DOCX 1611 kb)

## Abbreviations

COPD: Chronic obstructive pulmonary disease
CSE: Cigarette smoke extract
ERK1/2: Extracellular signal–regulated kinases
GM-CSF: Granulocyte-macrophage colony-stimulating factor
GRE: Glucocorticoid response element
GRα: Glucocorticoid receptor alpha
HBEC: Human bronchial epithelial cells
HDAC2: Histone deacetylase
ICS: Inhaled corticosteroids
LABA: Long-acting beta 2 agonists
LAMA: Long-acting muscarinic antagonist
LPS: Lipopolysaccharide
MIP3α: Macrophage Inflammatory Protein-3
MKP1: Mitogen-activated protein kinase phosphatase 1
MMP9: Matrix metallopeptidase 9
MUC1-CT: Mucin 1 cytoplasmic tail
PET: Positron emission tomography
SUV: Standard uptake value
TLR: Toll like receptor
