# Supplementary material for: MUC1 deficiency mediates corticosteroid resistance in chronic obstructive pulmonary disease
Source: Respir Res. 2018 Nov 20;19:226. doi: 10.1186/s12931-018-0927-4 (PMC6247701; doi:10.1186/s12931-018-0927-4)
Supplement: Supplementary file 1 — Figure S1. Acute cigarette smoke/ lipopolysaccharide lung inflammatory animal model showed resistance to dexamethasone improving lung resistance and bronchoalveolar inflammatory cell extravasation in Muc1 KO animals. Figure S2. IL-8 and IL-13 bronchoalveolar fluid content in Muc1 KO mice exposed to acute cigarette smoke/ lipopolysaccharide is resistant to dexamethasone. Figure S3. Inflammatory lung cell infiltration secondary to acute lipopolysaccharide/ cigarette smoke exposure is resistant to dexamethasone in MUC1 KO mice. (DOCX 1611 kb) [file 12931_2018_927_MOESM1_ESM.docx]

**ONLINE SUPPLEMENTARY MATERIAL**

**TITLE**

**MUC1 DEFICIENCY MEDIATES CORTICOSTEROID RESISTANCE IN CHRONIC OBSTRUCTIVE PULMONARY DISEASE**

**AUTHORS**

Javier Milara, Lucía Díaz-Platas, Sonia Contreras, Pilar Ribera, Inés Roger, Beatriz Ballester, Paula Montero, Ángel Cogolludo, Esteban Morcillo, Julio Cortijo

**SUPPLEMENTARY FIGURES**

**
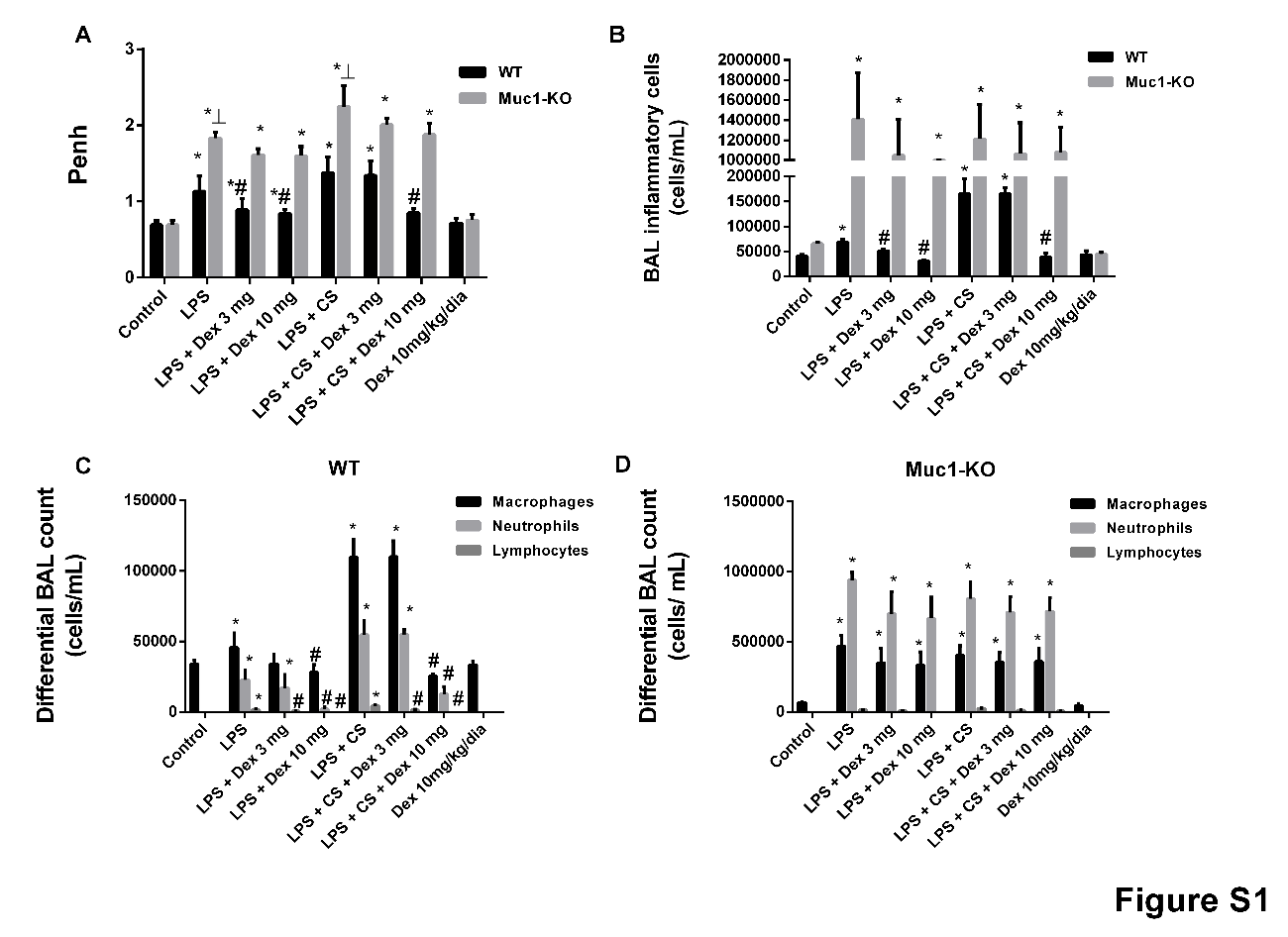
**

**Figure S1. Acute cigarette smoke/ lipopolysaccharide lung inflammatory animal model showed resistance to dexamethasone improving lung resistance and bronchoalveolar inflammatory cell extravasation in Muc1 KO animals.** C57BL/6 Muc1 KO mice and WT mice were undergoing intranasal instillation of 75μg of lipopolysaccharide (LPS) at day 1. Between days 2 and 3, animals were exposed to cigarette smoke (or control air) of 6 cigarettes followed by 8 cigarettes (or control air) at days 4 and 5, and 10 cigarettes (or control air) at day 6. Vehicle or dexamethasone at 3mg/kg/day and 10mg/kg/day was administered orally once a day between day 1 and 6. (a) Respiratory resistance (enhanced pause (Penh)) was measured at day 6 by plethysmography of whole body. (b)Total and (c,d) differential brochoalveolar (BAL) fluid inflammatory cells were analyzed in (c) WT and (d) Muc1 KO animals at day 6. Results are the mean ± SE of n = 8 animals per experimental group. One-way ANOVA followed by Bonferroni post-hoc tests. *p < 0.05 compared with control. #p<0.05 compared with LPS/ LPS+CS. ┴p<0.05 compared with WT. KO: knock out; WT: wild type; CS: cigarette smoke; LPS: lipopolysaccharide.


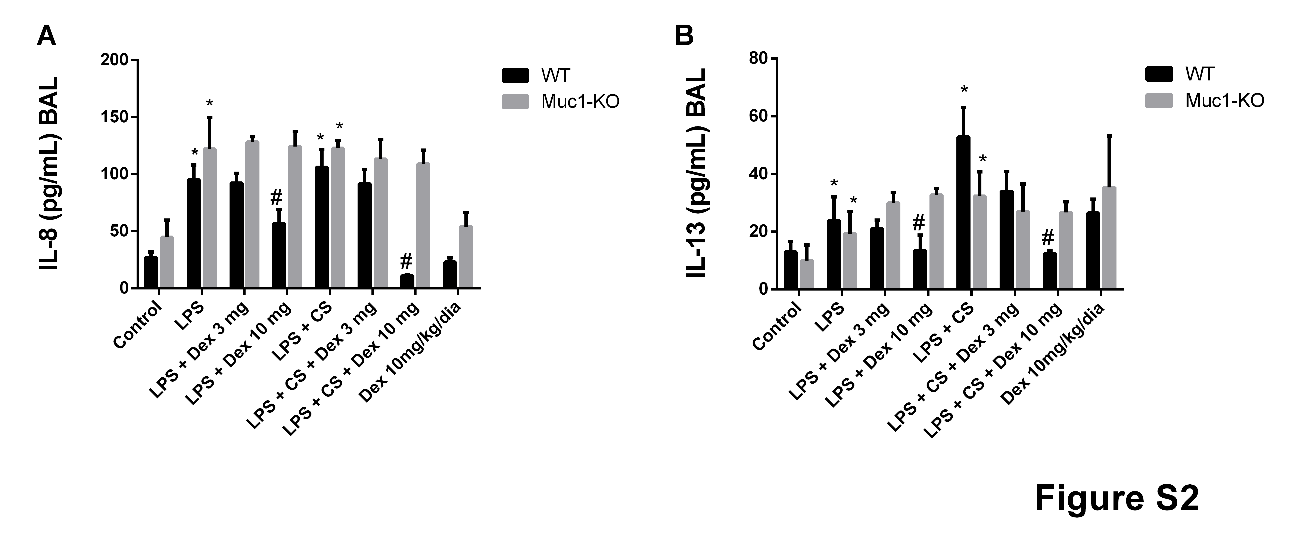


**Figure S2. IL-8 and IL-13 bronchoalveolar fluid content in Muc1 KO mice exposed to acute cigarette smoke/ lipopolysaccharide is resistant to dexamethasone.** C57BL/6 Muc1 KO mice and WT mice were undergoing intranasal instillation of 75μg of lipopolysaccharide (LPS) at day 1. Between days 2 and 3, animals were exposed to cigarette smoke (or control air) of 6 cigarettes followed by 8 cigarettes (or control air) at days 4 and 5, and 10 cigarettes (or control air) at day 6. Vehicle or dexamethasone at 3mg/kg/day and 10mg/kg/day was administered orally once a day between day 1 and 6. Bronchoalveolar (BAL) fluid content of IL-8 (a) and IL-13 (b) were measured by ELISA. Results are the mean ± SE of n = 8 animals per experimental group. One-way ANOVA followed by Bonferroni post-hoc tests. *p < 0.05 compared with control. #p<0.05 compared with LPS/ LPS+CS. KO: knock out; WT: wild type; CS: cigarette smoke; LPS: lipopolysaccharide.


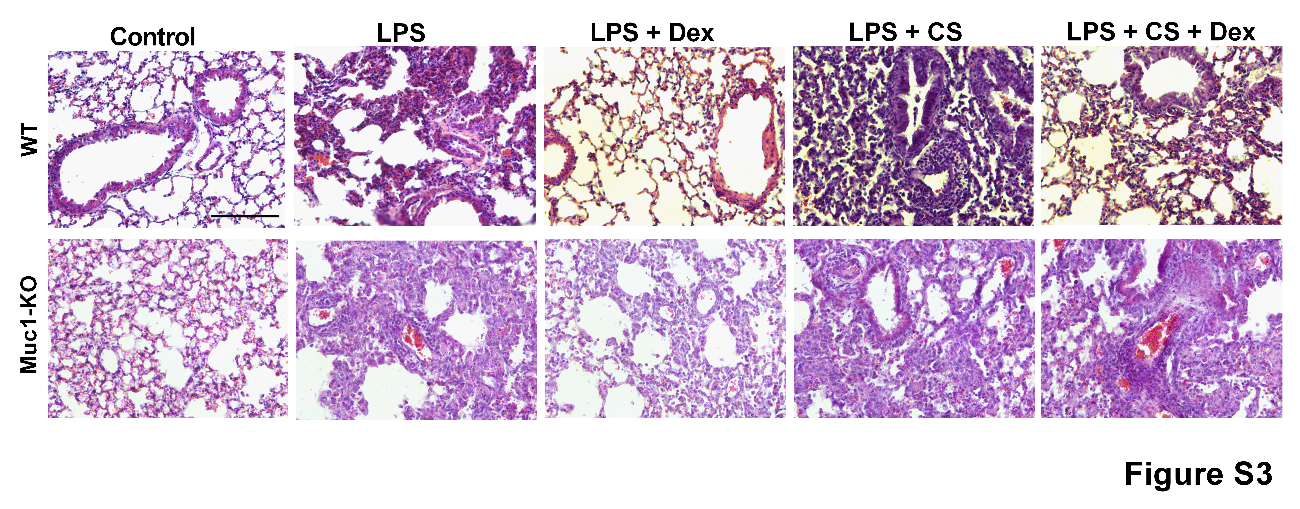


**Figure S3. Inflammatory lung cell infiltration secondary to acute lipopolysaccharide/ cigarette smoke exposure is resistant to dexamethasone in MUC1 KO mice.** C57BL/6 Muc1 KO mice and WT mice were undergoing intranasal instillation of 75μg of lipopolysaccharide (LPS) at day 1. Between days 2 and 3, animals were exposed to cigarette smoke (or control air) of 6 cigarettes followed by 8 cigarettes (or control air) at days 4 and 5, and 10 cigarettes (or control air) at day 6. Vehicle or dexamethasone at 3mg/kg/day and 10mg/kg/day was administered orally once a day between day 1 and 6. Hematoxiline & Eosine (H&E) histology was performed to evaluate inflammatory cell infiltration. Scale bar: 50µm. KO: knock out; WT: wild type; CS: cigarette smoke; LPS: lipopolysaccharide.
